# Dietary protein and blood pressure: an umbrella review of systematic reviews and evaluation of the evidence

**DOI:** 10.1007/s00394-024-03336-8

**Published:** 2024-02-20

**Authors:** Heiner Boeing, Anna M. Amini, Julia Haardt, Annemarie Schmidt, Heike A. Bischoff-Ferrari, Anette E. Buyken, Sarah Egert, Sabine Ellinger, Anja Kroke, Stefan Lorkowski, Sandrine Louis, Katharina Nimptsch, Matthias B. Schulze, Alexandra Schutkowski, Lukas Schwingshackl, Roswitha Siener, Armin Zittermann, Bernhard Watzl, Gabriele I. Stangl, the German Nutrition Society

**Affiliations:** 1 Department of Epidemiology (closed), German Institute of Human Nutrition Potsdam-Rehbruecke, Nuthetal, Germany; 2 German Nutrition Society, Godesberger Allee 136, 53175 Bonn, Germany; 3 Department of Aging Medicine and Aging Research, University Hospital Zurich, University of Zurich, and City Hospital Zurich, Zurich, Switzerland; 4 Institute of Nutrition, Consumption and Health, Faculty of Natural Sciences, Paderborn University, Paderborn, Germany; 5 Institute of Nutritional and Food Science, University of Bonn, Bonn, Germany; 6 Department of Nutritional, Food and Consumer Sciences, Fulda University of Applied Sciences, Fulda, Germany; 7 Institute of Nutritional Sciences, Friedrich Schiller University Jena, Jena, Germany; 8 Competence Cluster for Nutrition and Cardiovascular Health (nutriCARD), Halle-Jena-Leipzig, Germany; 9 Department of Physiology and Biochemistry of Nutrition, Max Rubner-Institut, Karlsruhe, Germany; 10 Molecular Epidemiology Research Group, Max Delbrück Center for Molecular Medicine (MDC) in the Helmholtz Association, Berlin, Germany; 11 Department of Molecular Epidemiology, German Institute of Human Nutrition Potsdam-Rehbruecke, Nuthetal, Germany; 12 Institute of Nutritional Science, University of Potsdam, Potsdam, Germany; 13 Institute of Agricultural and Nutritional Sciences, Martin Luther University Halle-Wittenberg, Halle (Saale), Germany; 14 Institute for Evidence in Medicine, Medical Center, Faculty of Medicine, University of Freiburg, Freiburg, Germany; 15 Department of Urology, University Stone Center, University Hospital Bonn, Bonn, Germany; 16 Clinic for Thoracic and Cardiovascular Surgery, Herz- und Diabeteszentrum Nordrhein Westfalen, Ruhr University Bochum, Bad Oeynhausen, Germany

**Keywords:** Dietary protein, Blood pressure, Hypertension, Umbrella review, Grading of evidence, Meta-analyses

## Abstract

**Introduction:**

This umbrella review aimed to investigate the evidence of an effect of dietary intake of total protein, animal and plant protein on blood pressure (BP), and hypertension (PROSPERO: CRD42018082395).

**Methods:**

PubMed, Embase and Cochrane Database were systematically searched for systematic reviews (SRs) of prospective studies with or without meta-analysis published between 05/2007 and 10/2022. The methodological quality and outcome-specific certainty of evidence were assessed by the AMSTAR 2 and NutriGrade tools, followed by an assessment of the overall certainty of evidence. SRs investigating specific protein sources are described in this review, but not included in the assessment of the overall certainty of evidence.

**Results:**

Sixteen SRs were considered eligible for the umbrella review. Ten of the SRs investigated total protein intake, six animal protein, six plant protein and four animal vs. plant protein. The majority of the SRs reported no associations or effects of total, animal and plant protein on BP (all “possible” evidence), whereby the uncertainty regarding the effects on BP was particularly high for plant protein. Two SRs addressing milk-derived protein showed a reduction in BP; in contrast, SRs investigating soy protein found no effect on BP. The outcome-specific certainty of evidence of the SRs was mostly rated as low.

**Discussion/conclusion:**

This umbrella review showed uncertainties whether there are any effects on BP from the intake of total protein, or animal or plant proteins, specifically. Based on data from two SRs with milk protein, it cannot be excluded that certain types of protein could favourably influence BP.

**Supplementary Information:**

The online version contains supplementary material available at 10.1007/s00394-024-03336-8.

## Introduction

Hypertension is an important modifiable risk factor for cardiovascular, cerebrovascular and chronic kidney diseases, and the leading underlying cause of global mortality and disability [1, 2]. It is suggested that 62% of cerebrovascular diseases and almost 50% of the ischaemic heart diseases are attributable to elevated BP, which will affect almost one-third of the adult population worldwide [2].

The American Heart Association categorised the systolic BP (SBP) and diastolic BP (DBP) into four ranges: normal (SBP < 120 mmHg and DBP < 80 mmHg), elevated (SBP 120 to 129 mmHg and DBP < 80 mmHg), stage 1 hypertension (SBP 130–139 mmHg or DBP 80 to 89 mmHg) and stage 2 hypertension (SBP ≥ 140 mmHg or DBP ≥ 90 mmHg) [3]. The regulation of BP is controlled by several complex mechanisms, such as baroreceptors, the activity of sympathetic nervous system, the renin–angiotensin–aldosterone system, antidiuretic hormone, natriuretic peptides and the nitric oxide system [4]. High BP which has emerged on the basis of medical conditions, such as renal diseases or endocrine disorders, is referred to as secondary hypertension. However, the most common form of hypertension is primary hypertension that is caused by a combination of genetic and lifestyle factors, such as obesity, physical inactivity, smoking and unhealthy diets [5]. In 2019, a network meta-analysis (MA) identified the Dietary Approaches to Stop Hypertension (DASH) diet, which favours a high intake of fruits and vegetables, low-fat dairy products, whole grains and low sodium, to be the most effective dietary strategy to reduce BP [6]. A recently published umbrella review, including 341 meta-analyses of randomised controlled trials (RCTs) and 70 meta-analysed observational studies, found high-quality evidence for a BP-lowering effect of the DASH diet. Additionally, the umbrella review demonstrated beneficial BP effects linked with the consumption of Mediterranean dietary patterns, which is characterised by low sodium, and moderate alcohol intake [7]. Notably, this umbrella review also included a few SRs on protein, revealing that high-protein diets were associated with BP-lowering effects in RCTs of low quality, but not in those of moderate quality. However, there is currently no published umbrella review focussing exclusively on the link between dietary proteins and BP.

Based on data showing that certain proteins may serve as a source of antihypertensive peptides [8], the hypothesis that dietary proteins can modulate BP appears biologically plausible. Most studies in the field of bioactive peptides have been published on milk peptides; among them, several peptides have been identified which can inhibit the angiotensin-converting enzyme (ACE) and lower BP [9, 10]. In addition to peptides, certain amino acids (AS) have been linked with mechanisms controlling BP and beneficial effects on elevated BP. For example, an MA of RCTs on the effect of L-arginine supplementation demonstrated a significant reduction in SBP of − 6.40 mmHg and DBP of − 2.64 mmHg and identified the effective dosage of L-arginine for SBP reduction to be ≥ 4 g per day [11].

It is therefore tempting to speculate that the intake of high-protein diets or proteins from plant and/or animal origin can modulate BP. The current umbrella review addressed the level and certainty of evidence derived from SRs concerning whether dietary intake of protein, and proteins from plant and animal sources in general are capable of modifying BP or hypertension risk in the general adult population. Further, proteins from specific food sources were also reviewed but not evaluated for evidence. The present umbrella review will contribute to the upcoming evidence-based guideline for protein intake of the German Nutrition Society considering different pathologies.

## Methods

We conducted an umbrella review (PROSPERO: CRD42018082395) following the methodological protocol published by Kroke et al. [12]. This protocol was developed as part of the evaluation of protein intake and various health-related outcomes and was also used for BP. In preparing this manuscript, we followed the guidelines of reporting outlined in the PRISMA 2020 checklist [13]. The literature search, selection of SRs, data extraction and evaluation of the methodological quality and outcome-specific certainty of evidence was conducted independently by two authors (AMA, AnS). Any disagreements were resolved by discussion to reach consensus.

### Literature search

The systematic literature search was conducted in PubMed, Embase and Cochrane Database of Systematic Reviews for SRs published between 05/2007 and 10/2022 to cover a period of at least 10 years. The initial database search was conducted in 05/2017 and was updated on 6 October 2022 due to elapsed time reasons. The search strategies regarding study type (SRs), proteins (exposure or intervention) and BP in general, as well as SBP, DBP and hypertension, are presented in Supplementary Material S1. In addition to the SRs found in this context, reference lists of included SRs were screened for further SRs of relevance.

### Literature selection

Titles and/or abstracts of the results of the literature searches were screened according to pre-defined inclusion and exclusion criteria [12] in order to identify potentially eligible SRs. The full texts of potentially eligible records were retrieved and assessed for final eligibility.

SRs had to address the general adult population (without lactating women or top athletes) as inclusion criteria and were eligible for the umbrella review if they analysed one of the following study designs: SR with or without MA of prospective studies in humans (RCTs, prospective cohort studies, case-cohort studies or nested case–control studies). If an SR also included case–control studies or cross-sectional studies, those studies or MAs predominantly including those studies (≥ 50% of all studies) were not considered. The SRs had to address the association/effect between protein intake and SBP, DBP or the incidence of hypertension. All SRs that exclusively meta-analysed studies with whole foods were excluded. From SRs addressing studies with whole foods, only the studies which addressed proteins were considered in this umbrella review.

### Data extraction

The following data from each included SR were extracted: the first author’s surname, year of publication, study type (e.g. SR with MA of RCTs), study duration(s), study population, intervention/exposure(s), outcome(s), effect estimate(s) including 95% confidence intervals (CIs), p-value(s) and heterogeneity estimate(s). Corresponding and first authors were contacted in case of insufficient data. Where results were reported from multiple analysis methods (e.g. MA conducted with both end of study and change values), we extracted all available results into Table 1. Subsequently, for the purpose of rating the overall certainty of evidence, content experts (HB and GIS) determined the selection of data to be utilised. Further, the utilised single original studies in each SR are listed in Supplementary Material S2, subdivided by study type and intervention/exposure.

**Table 1:** Characteristics of the included systematic reviews

References	Study type, study duration/follow-up	Study population	Exposition	Protein intake	Outcome	Pooled effect estimates (95% CI)	Heterogeneity estimator	NutriGrade rating	AMSTAR 2 rating
*(A) Total protein studies*									
Rebholz et al. [19]	SR with MA of 29 RCTs RCTs published between 1980 and 04/11 Study duration: 1–24 wk	*n* = 2546 Both sexes Mean age: 26–62 yr (age range: 18–80 yr)	Protein vs. carbohydrates	Supplement intervention studies: 20–54 g/d vs. 0–8 g/d (control dose NP for all studies) Feeding/diet intervention studies: 19.5–95.0 En% vs. 11.5–50.0 En%	SBP	Net change: − 1.76 mmHg (− 2.33, − 1.20) *P* < 0.001	*I*^2^ = 0%	Moderate	Low
					DBP	Net change: − 1.15 mmHg (− 1.59, − 0.71) *P* < 0.001	*I*^2^ = 0%	Moderate	
Santesso et al. [20]	SR with MA of RCTs RCTs published before 08/11 Study duration: 28–365 d	Both sexes Mean age: 26–54 yr Healthy or with hypertension, overweight, obesity, hyperlipidemia or metabolic syndrome	Higher- vs. lower-protein diets	25–35 En% vs. 15–24 En%					High
	MA conducted with end of study values: 21 RCTs	*n* = 1337			SBP	SMD: − 0.07 (− 0.21, 0.07) *P* = 0.33	*I*^2^ = 24%	Low	
					DBP	SMD: − 0.03 (− 0.15, 0.09) *P* = 0.63	*I*^2^ = 9%	Low	
	MA conducted with change values: 15 RCTs	*n* = 1186			SBP	SMD: − 0.21 (− 0.32, − 0.09) *P* = 0.0004	*I*^2^ = 0%		
					DBP	SMD: − 0.18 (− 0.29, − 0.06) *P* = 0.003	*I*^2^ = 2%		
Wycherley et al. [21]	SR with MA of 5 RCTs RCTs published before 05/11 Study duration: 4–16 wk	*n* = 230 Both sexes Mean age: 26 to ~ 62 yr Healthy or with overweight, obesity, type 2 diabetes or polycystic ovary syndrome	(High-protein, energy-restricted, low-fat diet) vs. (standard-protein, energy-restricted, low-fat diet) at least 10% difference in protein intake	28–33 En% (1.14–1.40 g/kg BW) vs. 16–21 En% (0.68–0.88 g/kg BW), this info was only provided for 4 out of 5 studies	SBP	WMD: − 2.09 mmHg (− 5.01, 0.83) *P* = 0.16	*I*^2^ = 0%	Low	Low
					DBP	WMD: − 0.72 mmHg (− 2.67, 1.23) *P* = 0.47	*I*^2^ = 0%	Low	
Pedersen et al. [22]	SR of 4 studies ( 1 RCTs, 2 cohort studies and 1 SR with MA) Studies published between 01/00 and 12/11 Study duration: NP	*n* = 41,170 Both sexes Healthy or with prehypertension or stage 1 hypertension	Total protein	This info was only available for 1 out of the 4 included studies: 25 vs. 15 En%	SBP, DBP and hypertension	All studies: non-significant association/effect	NA	Low^a^: SBP and DBP Moderate: hypertension	Moderate
Schwingshackl and Hoffmann [23]	SR with MA of 11 RCTs RCTs published before 08/12 Study duration: 1–2 yr	*n* = 1414 (SBP) *n* = 1402 (DBP) Both sexes Mean age: 41 to > 60 yr Healthy or with type 2 diabetes	High protein (≥ 25 En%) vs. low protein (≤ 20 En%) with both low fat (≤ 30 En%)	25–40 En% vs. 10–20 En%	SBP	WMD: − 1.61 mmHg (− 3.45, 0.23) *P* = 0.09	*I*^2^ = 41%	Low	High
					DBP	WMD: − 0.42 mmHg (− 1.37, 0.54) *P* = 0.39	*I*^2^ = 0%	Low	
Clifton et al. [24]	SR with MA of 19 weight loss/maintenance RCTs RCTs published before 08/13 Study duration: 52–104 wk	*n* = 2650 Both sexes Mean age: 40–63 yr Healthy or with type 2 diabetes or polycystic ovary syndrome	High protein, low carbohydrate weight loss diet vs. control diet	25–60 En% vs. 10–30 En%	SBP	SMD: − 0.022 (− 0.13, 0.09) *P* = 0.69	*I*^2^ = 33%	Low	Moderate
					DBP	SMD: 0.11 OR − 0.062 (− 0.23, 0.01) *P* = 0.104 OR 0.08^b^	*I*^2^ = 52%	Low	
Mousavi et al. [25]	SR with MA of 5 cohort studies Studies published until 04/20 Follow-up: 2–11 yr	*n* = 93,496 (5,620 cases) Both sexes Age range: 18–65 yr General population	Total protein	NP		RR: 1.01 (0.90, 1.14) dose–response analysis (3 cohort studies): RR: 0.99 (0.83, 1.18) per 5% increase in energy intake from total protein P_linear_ = 0.96	*I*^2^ = 47%	Moderate	Moderate
Lonnie et al. [26]	SR without MA of 1 RCT RCT published before 03/20 Study duration: 3 wk	*n* = 48 Both sexes Mean age 58 yr With hypertension and overweight/obesity	Protein mix (pea protein, soya protein, milk protein and egg white protein) vs. maltodextrin *vs*. sucrose		Postprandial SBP	Lower SBP with maltodextrin compared to protein mix No difference in SBP with sucrose compared to protein mix	NA	Very low	Low
					Postprandial DBP	Lower DBP with maltodextrin compared to protein mix No difference in DBP with sucrose compared to protein mix	NA	Very low	
Vogtschmidt et al. [27]	SR with MA of 25 RCTs RCTs published before 11/20 Study duration: 4–52 wk	*n* = 1813 Both sexes Mean age: 26–71 yr Healthy or with prehypertension, hypertension, overweight, obesity, insulin resistance, hyperlipidemia, hyperinsulinemia or polycystic ovary syndrome	Higher- vs. lower-protein diets (hypo- and isocaloric)	20–36 En% vs. 14–23 En%	SBP	SMD: − 0.12 (− 0.21, − 0.02) *P* NP	*I*^2^ = 0%	Low	High
					DBP	SMD: − 0.09 (− 0.19, 0.01) *P* not statistically significant	*I*^2^ = 9%	Low	
					SBP	WMD: − 1.16 mmHg (− 2.13, − 0.20) *P* NP	*I*^2^ = 0%	Low	
Hengeveld et al. [28]	SR without MA of 4 RCTs RCTs published before 04/20 Study duration: 12 wk–2 yr	*n* = 312 Both sexes Mean age: 67–74 yr Older adults from the general population Healthy or with overweight, obesity or sarcopenia	High protein vs. low Protein (2 RCTs with concomitant exercise in control and intervention group)	1.0–1.5 g/kg BW/d vs. 0.8–1.1 g/kg BW/d	SBP	None of the RCTs showed an effect	NA	Low	Moderate
					DBP	None of the RCTs showed an effect	NA	Low	
*(B) Animal protein studies*									
Rebholz et al. [19]	SR with MA of 15 RCTs RCTs published between 1980 and 04/11 Study duration: NP	*n* = NP Both sexes Mean age: NP	Animal protein (meat, fish, poultry, milk, casein or whey) vs. carbohydrates	Supplement intervention studies: 24.4–54.0 g/d vs. 0–2.8 g/d (control dose NP for all studies) Feeding/diet intervention studies: 19.5–95.0 En% vs. 15.2–50.0 En%	SBP	Net change: − 2.54 mmHg (− 3.55, − 1.53) *P* < 0.001	I^2^ = 0%	Moderate	Low
					DBP	Net change: − 0.95 mmHg (− 1.72, − 0.19) *P* = 0.014	I^2^ = 0%	Moderate	
Pedersen et al. [22]	SR of 2 cohort studies Studies published between 01/00 and 12/11 Study duration: NP	*n* = 7594 Both sexes Healthy	Animal protein	NP	SBP, DBP and hypertension	All studies: non− significant association	NA	Low: SBP, Low: DBP, Moderate: hypertension	Moderate
Chalvon-Demersay et al. [37]	SR without MA of 4 prospective studies Studies published before 03/16 Follow-up: 1.5–11 yr	*n* = 4157 Both sexes Mean age: 42–70 yr Healthy or with hypertension, overweight, obesity, diabetes or hypercholesteremia	Animal protein	NP	SBP & DBP	3 studies: no effect with animal protein 1 study: inverse relation between animal protein and BP	NA	Low^c^	Moderate
Hidayat et al. [35]^d^	SR with MA of 7 RCTs RCTs published before 05/16 Study duration: 4 wk–2 yr	*n* = 412 Both sexes Mean age: 23–61 yr, Healthy or with prehypertension, overweight, obesity, hypercholesterolemia or metabolic syndrome	Milk protein (low-fat milk, whey protein isolate, whey or casein, reduced-fat fortified milk or whey protein isolate and sodium caseinate) vs. (carbohydrates, usual diet, no supplementation or placebo)	70 mg–82 g extra milk protein/d	SBP	WMD: − 3.33 mmHg (− 5.62, − 1.03) *P* NP	*I*^2^ = 0%	Low	High
					DBP	WMD: − 1.08 mmHg (− 3.38, − 0.22) *P* NP	*I*^2^ = 0%	Low	
Badely et al. [36]^e^	SR with MA of 18 RCTs Literature search period: NP Study duration: 4–96 wk	*n* = 885 Both sexes Mean age: 23–74 yr With overweight or obesity	Whey protein (powder, supplement, isolate, hydrolyse, concentrate) vs. food protein, soy protein, (calcium) casein, casein + carbohydrates, glucose + lycopene, soy, low-fat conventional yogurt, placebo, control diet	0.7–110 g whey protein/d vs. 0–110 g non-whey protein/d	SBP	WMD: − 7.76 mmHg (− 9.39, − 6.14) *P* < 0.001	*I*^2^ = 100%	Low	Low
					DBP	WMD: − 5.69 mmHg (− 6.70, − 4.68) *P* < 0.001	*I*^2^ = 99%	Low	
Mousavi et al. [25]	SR with MA of 5 cohort studies Studies published until 04/20 Follow-up: 2–11 yr	*n* = 93,496 (5,620 cases) Both sexes Age range: 18–65 yr General population	Animal protein		Hypertension	RR: 1.03 (0.89, 1.19) Dose–response analysis (2 cohort studies): RR: 0.92 (0.60, 1.42) per 5% increase in energy intake from animal protein	*I*^2^ = 68%	Moderate	Moderate
*(C) Plant protein studies*									
Rebholz et al. [19]	SR with MA of 9 RCTs RCTs published between 1980 and 04/11 Study duration: NP	*n* = NP Both sexes Mean age: NP	vegetable protein (soy or other plant protein) vs. carbohydrates	Supplement intervention studies: 20–40 g/d vs. 0–8 g/d (control dose NP for all studies) feeding studies: 22.8–23.7 En% vs. 11.5–12.2 En%	SBP	Net change: − 2.27 mmHg (− 3.36, − 1.18) *P* < 0.001	*I*^2^ = 5%	Moderate	Low
					DBP	Net change: − 1.26 mmHg (− 2.26, − 0.26) *P* = 0.014	*I*^2^ = 22%	Moderate	
Pedersen et al. [22]	SR of 3 studies (2 cohort studies and 1 SR with MA) Studies published between 01/00 and 12/11 Study duration: NP	*n* = 9202 Both sexes Healthy or with prehypertension or stage 1 hypertension	Plant protein	This info was only available for 1 out of the 3 included studies: 18–66 g extra soy protein/d	SBP, DBP and hypertension	All studies: inverse association	NA	Low: SBP Low: DBP High: hypertension	Moderate
Chalvon-Demersay et al. [37]	SR without MA of 4 prospective studies Studies published before 03/16 Follow-up: 1.5–11 yr	*n* = 4157 Both sexes Mean age: 42–70 yr Healthy or with hypertension, overweight, obesity, diabetes or hypercholesterolemia	Plant protein	NP	SBP and DBP	All studies: inverse relation between plant protein and BP	NA	Moderate^f^	Moderate
Mousavi et al. [25]	SR with MA of 5 cohort studies Studies published until 04/20 Follow-up: 2–11 yr	*n* = 93,496 (5620 cases) Both sexes Age range: 18–65 yr General population	Plant protein	NP	Hypertension	RR: 0.87 (0.74, 1.01) Dose–response analysis (2 cohort studies): RR: 0.76 (0.65, 0.90) per 5% increase in energy intake from plant protein	*I*^2^ = 68%	Moderate	Moderate
Mohammadifard et al. [38]^g^	SR with MA of 2 RCTs RCTs published before 06/20 Study duration: 8–12 wk	*n* = 92 Females only Mean age: 48–64 yr With metabolic syndrome	Soya protein vs. control (“control” or DASH diet)	30–35 g/d	SBP	WMD: − 0.04 mmHg (− 0.33, 0.25)	*I*^2^ = 62%	Very low	Low
					DBP	WMD: 0.13 mmHg (− 0.16, 0.42)	*I*^2^ = 25%	Very low	
Mosallanezhad et al. [39]^h^	SR without MA of 4 RCTs RCTs published before 06/20 Study duration: 5–24 wk	*n* = 605 Both sexes Mean age: 51–61 yr Healthy or with hypertension or hypercholesterolemia	Soya protein vs. control	NP	SBP	2 out of 4 comparisons: no difference between intervention and control 2 out of 4 comparisons: lower SBP with soya protein	NA	Moderate	Moderate
					DBP	2 out of 4 comparisons: no difference between intervention and control 2 out of 4 comparisons: lower DBP with soya protein	NA	Moderate	
*(D) Animal *vs.* plant protein studies*									
Rebholz et al. [19]	SR with MA of 12 RCTs RCTs published between 1980 and 04/11 Study duration: 4–12 wk	*n* = 870 Both sexes Mean age: 36–65 yr (age range: 18–79 yr)	Vegetable protein (soy or other plant protein) vs. animal protein (meat, fish, poultry, milk, casein or whey)	Supplement intervention studies: 30–50 g/d vs. 40–50 g/d (control dose NP for all studies) Feeding/diet intervention studies: 12.6–21.2 En% vs. 12.9–20.7 En%	SBP	Net change: − 0.10 mmHg (− 2.31, 2.11) *P* NP	*I*^2^ = 97%	Low	Low
					DBP	Net change: − 0.24 mmHg (− 1.58, 1.10) *P* NP	*I*^2^ = 78%	Low	
Chalvon-Demersay et al. [37]	SR without MA of 10 RCTs RCTs published before 03/16 Study duration: 4 wk–1 yr	*n* = 1199 Both sexes Mean age: 21–68 yr Healthy or with prehypertension, stage 1 hypertension, overweight, obesity or hypercholesterolemia	Animal vs. plant protein	15–90 g (additional) protein/d	SBP and DBP	7 RCTs: no differential effect of plant− vs. animal− sourced proteins 3 RCTs: inconsistent effects	NA	Moderate	Moderate
Lonnie et al. [26]	SR without MA of 3 RCTs RCT s published before 03/20 Study duration: 1 d–8 wk	*n* = 105 Both sexes Age range: 18–80 yr Healthy or with hypertension, overweight, obesity or hypercholesterolemia	Extracted plant protein (lupin or pea isolate) vs. animal protein (milk and/or egg white)	25 g/d or 0.6 g/kg BW/d	SBP	3 out of 4 comparisons: no difference between intervention and control 1 out of 4 comparisons: higher SBP with egg white protein compared to pea protein	NA	Low	Low
					DBP	3 out of 4 comparisons: no difference between intervention and control 1 out of 4 comparisons: higher DBP with egg white protein compared to pea protein	NA	Low	
Bryant et al. [40]	SR without MA of 2 RCTs RCTs published between 01/00 and 09/21 Study duration: 28 d–8 wk	*n* = 101 Both sexes Mean age: 49–60 yrs All with hypercholesterolemia	Lupin protein isolate vs. milk protein isolate (isocaloric)	25 g/d	SBP	Both RCTs: no differences between intervention and control	NA	Low	Moderate
					DBP	Both RCTs: no differences between intervention and control	NA	Low	

*AMSTAR 2* A Measurement Tool to Assess Systematic Reviews 2, *BP* blood pressure, *BW* body weight, *CI* confidence interval, *d* day(s), *DBP* diastolic blood pressure, *En*% percentage of energy intake, *MA* meta-analysis, *mmHg* milimetre of mercury, *NA* not applicable, *NP* not provided, *RCT* randomised controlled trial, *RR* relative risk, *SBP* systolic blood pressure, *SMD* standardised mean difference, *SR* systematic review, wk week(s), *WMD* weighted mean difference, *yr* year(s)

^a^A total of 4 NutriGrade assessments was conducted and all received a rating of low: RCTs: SBP and total protein, RCTs: DBP and total protein, cohorts: SBP and total protein, cohorts: DBP and total protein

^b^The manuscript states two different values for the effect estimator and the *P* value. The author was contacted for clarification, but no answer was received

^c^A total of 2 NutriGrade assessments were conducted (DBP and animal protein and SBP and animal protein), both received a rating of low

^d^This SR does not address animal protein in general, but specific protein sources and was therefore not used to assess the overall certainty of evidence

^e^This SR does not adress animal protein in general, but specific protein sources and was therefore not used to assess the overall certainty of evidence

^f^A total of 2 NutriGrade assessments was conducted (DBP and plant protein and SBP and plant protein), both received a rating of moderate

^g^This SR does not address plant protein in general, but specific protein sources and was therefore not used to assess the overall certainty of evidence

^h^This SR does not address plant protein in general, but specific protein sources and was therefore not used to assess the overall certainty of evidence

### Assessments of methodological quality and outcome-specific certainty of evidence

The methodological quality of included SRs was assessed using a modified version of the “A Measurement Tool to Assess Systematic Reviews 2” tool (AMSTAR 2) [14], and the modifications are described in detail in our methodological protocol [12]. This version contains 14 evaluation items grading the methodology of SRs on a scale from high quality to critically low quality according to the presence of critical and non-critical methodological weaknesses (Supplementary Material S3). SRs graded as "critically low" by AMSTAR 2 were excluded from the evaluation of the overall certainty of evidence. The outcome-specific certainty of evidence of included SRs with and without MA was assessed using the NutriGrade scoring tool [15] (Supplementary Material S4). It utilises a numerical scoring system, and four categories rate the potential outcome-specific certainty of evidence: high, moderate, low and very low. The NutriGrade scoring tool was modified for the assessment of SRs without MA, and the adaptions are described in detail elsewhere [12]. For SRs reporting more than one relevant exposure or outcome, a separate assessment by NutriGrade was conducted.

### Rating of the overall certainty of evidence

The overall certainty of the evidence was assessed according to Kroke et al. [12] and is described in Supplementary Material S5. Kroke et al. [12] proposed using specific criteria for grading, including result concordance, existing biological plausibility, methodological quality and outcome-specific certainty of evidence. The assessment was performed for the intake of total protein, as well as proteins derived from animal and plant sources. SRs which addressed specific protein sources, but not protein intake or animal and plant proteins in general, were included in this review, but not used to assess the overall certainty of evidence. The rating of the overall certainty of evidence was conducted independently by three authors (HB, AMA, GIS). Any disagreements were resolved by discussion to reach consensus.

## Results

**Fig. 1:**
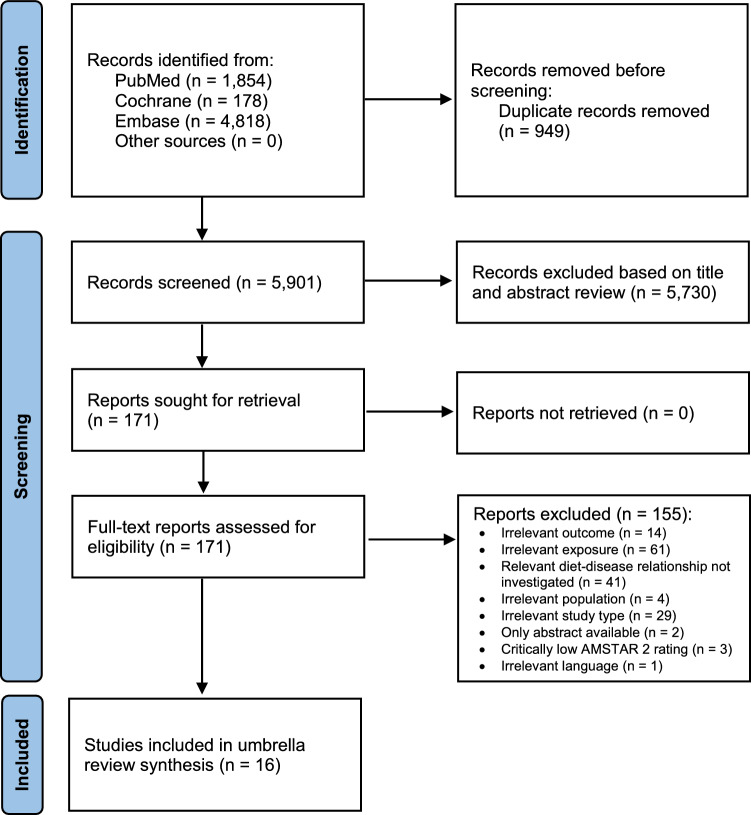
Flow diagram

The study selection process is outlined in the flow diagram depicted in Fig. 1. The literature search within the three databases identified 6850 potentially eligible publications, which were reduced to 5901 articles when duplicates were removed. 5730 publications were excluded due to irrelevant titles and abstracts. In total, 171 articles were subjected to full text screening. Out of the 171, 155 were found not to be eligible due to different reasons, which are shown in Supplementary Material S6. Most of the reasons of non-eligibility referred to irrelevant exposures or lack of exposure-outcome investigations fitting the research question. Three SRs were excluded because of a “critically low” AMSTAR 2 rating [16–18] (Supplementary Material S7). All three SRs used only one database for their literature search. Additionally, Altorf-van der Kuil et al. [16] and Tielemans et al. [18] failed to conduct an adequate risk of bias assessment and Dong et al. [17] failed to provide a list of excluded studies. A total of 16 SRs were considered eligible to be addressed in this umbrella review. Details of these studies (outcomes, rating according to methodological quality, outcome-specific certainty of evidence) are found in Table 1, which subdivides the SRs into total protein, animal and plant protein and those which compared animal with plant proteins. The detailed results of the assessment of the methodological quality are shown in Supplementary Material S7 and of the outcome-specific certainty of evidence in Supplementary Material S8. The duration of the underlying primary RCTs ranged from one week to two years. Approximately 10% had a duration of one to four weeks, while around 20% had a study duration of at least one year. The sample size of these underlying primary RCTs ranged between seven and 419 participants, with approximately 10% having fewer than 22 participants, around 20% having more than 100 participants, and approximately 10% having more than 150 participants. There were only eight underlying primary cohort studies, with a follow-up duration of 1.5–11.3 years. Their sample size ranged from 272 to 80,426 participants. Two cohort studies investigated fewer than 1000 participants, while five cohort studies had participant numbers ranging between 1361 and 5880. One cohort study had a relatively large number of participants, specifically 80,426.

### Total protein studies

Ten SRs addressed total protein intake and BP [19–28] (Table 1A). Two of these SRs included prospective cohort studies and investigated the association between total protein intake and BP or hypertension [22, 25]. The other SRs analysed only RCTs [19–21, 23, 24, 26–28]. The SRs with RCTs usually included individuals of both sexes and healthy subjects, but also accepted original studies that were conducted with individuals with overweight, hypertension and diabetes. High-protein diets used in the RCTs typically contained more than 25 energy% (*E*%) proteins. The quantity of proteins in the control diets mostly ranged from 10 to 20 *E*%. The study of Rebholz et al. [19] analysed only RCTs in which carbohydrates were replaced by protein.

The two SRs including prospective studies did not find associations between protein and BP or hypertension [22]. The SR of Pedersen et al. [22] included two prospective cohort studies [29, 30], but also a SR with MA [31], that analysed cross-sectional studies, but also two prospective cohort studies, one with young adults [32] and another with children [33]. The prospective cohort study with young adults found inverse, but predominantly non-significant associations between protein intake and BP [32]. The SR of Mousavi et al. [25] meta-analysed five cohort studies and did not find a significant association between total protein intake and risk of hypertension.

Nine SRs of RCT reviewed or meta-analysed the effect of total protein intake on BP [19–24, 26–28]. Rebholz et al. [19] found a reduction in SBP and DBP when carbohydrates were replaced by protein. The SR of Santesso et al. [20] observed no effect of protein on BP when comparing the final values between the intervention and control group (21 RCTs), but reported a significant reduction in BP following protein intake (15 RCTs) when comparing the final and baseline values. The SR of Wycherley et al. included five RCTs on BP and did not find significant effects of high-protein diets on SBP and DBP [21]. Schwingshackl and Hoffmann [23], conducting an MA of 11 RCTs addressing a similar research question, reached the same conclusion, revealing no effect of protein intake on SBP or DBP, neither in the MA of all RCTs, nor in the subgroup of high-quality RCTs [23]. No conclusive effects of dietary proteins on BP were found in the SR of Pedersen et al. [22]. An insufficient control of ethnicity and body weight were given as reasons for this conclusion [22]. Clifton et al., who meta-analysed 19 long-term (> 12 months) weight loss RCTs, did not observe a significant effect of dietary protein in exchange for carbohydrates on SBP and DBP [24]. The SR of Lonnie et al. [26] included only one RCT that investigated the effect of total protein (mixture of pea protein, soy protein, milk protein, egg white protein) compared to maltodextrin or sucrose on postprandial BP [34]. This study found no protein effect on postprandial SBP. In contrast, DBP was significantly increased 60 min postprandial compared to maltodextrin, but not compared to sucrose intervention. The SR of Vogtschmidt et al. [27] included an MA of 25 RCTs and found high-protein diets (protein range: 20–36 E%) accompanied by a greater reduction in SBP than low-protein diets (14–23 *E*%), whereas the protein effect on DBP did not reach statistical significance. The SR of Hengeveld et al. [28], included four RCTs on protein intake and BP. None of these studies found an effect of an increased protein intake on SBP and DBP. The authors remarked critically that three of the four RCTs did not reach a sufficient statistical power to demonstrate an effect on BP.

The methodological quality assessed with AMSTAR 2 was graded as high for three SRs (all SRs of RCTs) [20, 23, 27], moderate for four SRs [22, 24, 25, 28] and low for three SRs [19, 21, 26] (Table 1A). The methodological quality of the SRs was independent of the publication date. The NutriGrade assessment included 23 entries separated according to the outcome investigated. Most of the ratings regarding the outcome-specific certainty of the evidence were low (*n* = 17). Only four exposure-outcome assessments were rated as moderate, and two assessments as very low. The list of studies being used in the SRs that demonstrate the potential study overlap is shown in Supplementary Material S2. The majority of RCTs on total protein were used also once (*n* = 44), while 29 RCTs were utilised multiple times (up to five times), mostly published between 2000 and 2005. Regarding cohort studies, there was only minor overlap (Supplementary Material S2B).

### Animal protein studies

Six SRs addressed animal protein and BP, with two of them analysing RCTs with milk proteins [35, 36]. Of the remaining four SRs on animal protein, three included cohort studies [22, 25, 37], and one analysed RCTs that replaced carbohydrates by animal protein [19]. Pedersen et al. [22] who analysed two cohort studies did not find an association between animal protein intake and BP. The SR of Chalvon-Demersay et al. [37] found in three out of four prospective studies no link between animal protein and BP, and an inverse association in one study. The formally well-performed quantitative SR with MA of Mousavi et al. [25] including five cohort studies did not find animal protein intake associated with the risk of hypertension. The only SR with RCTs specifically addressing animal protein found that animal protein replacing carbohydrates led to significant reductions in SBP and DBP [19]. There are also two SRs of RCTs that addressed subtypes of animal proteins, in particular, proteins from milk. These were the SR of Hidayat et al. [35], who meta-analysed seven RCTs that investigated the effect of milk protein, in particular, whey protein and casein, on BP, and the SR of Badely et al. [36], who meta-analysed 18 RCTs that investigated the effect of whey protein on BP. Both SRs found a reduction in SBP and DBP following milk protein intake. The findings of these SRs are important with respect to health implications and dietary recommendations, but are not within the scope of the current review that aimed to investigate animal proteins in general. Thus, both SRs were not included in the evaluation of the overall certainty of evidence.

The outcome-specific certainty of evidence (NutriGrade rating) was rated four times as moderate and eight times as low, and the methodological quality (AMSTAR 2 rating) once as high [35], three times as moderate [22, 25, 37] and twice as low [19, 36]. The list of original studies being used in the SRs is shown in Supplementary Material S2C and S2D. The studies used by Pedersen et al. [22] were also used by Chalvon-Demersay et al. [37] and Mousavi et al. [25], but there was no overlap of studies in the SRs of Chalvon-Demersay et al. [37] and Mousavi et al. [25].

### Plant protein studies

Six SRs addressed plant proteins and BP [19, 22, 25, 37–39] (Table 1C). One SR analysed RCTs that replaced carbohydrates by plant proteins [19], three SRs analysed observational studies including cohort studies [22, 25, 37] and two SRs analysed soy products which included also studies with soy protein [38, 39]. According to the procedure of SRs with milk proteins, SRs which exclusively addressed soy were not used for the evaluation of the overall certainty of evidence of plant proteins on BP. Pedersen et al. [22] concluded that their SR provided evidence for an inverse relationship between plant protein and BP. This conclusion was based on two cohort studies, that found an inverse relationship between plant protein and BP and a MA of RCTs addressing soy protein, which show a BP-lowering effect of these plant proteins [17]. This MA was excluded from this umbrella review due to its low methodological quality [17]. In the SR of Chalvon-Demersay et al. [37], the four cohort studies showed an inverse relationship between plant protein intake and SBP and DBP, respectively. Mousavi et al. [25] concluded in their SR that plant protein intake was not associated with risk of hypertension, although a subset of dose–response studies observed an inverse relationship between plant proteins and BP. Rebholz et al. [19] observed an inverse relation in RCTs when carbohydrates were replaced by plant proteins.

In the SR of Mohammadifard et al. [38] which addressed health effects of soy in subjects with the clinical diagnosis of metabolic syndrome, only two RCTs focussed on the effects of soy protein on BP. These two RCTs did not find any effect of soy protein on SBP and DBP, and were in line with the overall findings of consumption of soy products on BP in this SR. The SR on soy conducted by Mosallanezhad et al. included four RCTs on soy protein [39], two of them showed a BP-lowering effect and the other two observed no effect on BP.

The outcome-specific certainty of evidence was rated twice as very low, two times as low, seven times as moderate and once as high. The methodological quality was considered to be moderate in three [22, 25, 37] of the four SRs and low in one [19]. There had been a moderate overlap of studies included in the SRs (Supplementary Material S2E and S2F).

### RCTs with animal vs. plant protein

Four eligible SRs (Table 1D) included studies that compared the effects of animal proteins with plant protein intake on BP [19, 26, 37, 40]. The SR of Rebholz et al. [19] included 12 RCTs that compared plant with animal proteins on BP, but found no differences between these two protein sources. No differences between plant and animal proteins on BP were observed in the SR of Chalvon-Demersay et al. [37] either, which included 10 RCTs, most of them with soy protein as the plant protein source. The SR of Lonnie et al. [26] included three relevant RCTs, two of them did not show differences between animal and plant proteins on SBP and DBP, and one RCT found higher postprandial BP values following egg white protein consumption compared to plant protein intake. Bryant et al. [40] analysed two RCTs in their SR on subjects with hypercholesterolemia and found that lupin protein isolates and milk protein did not differ in their effect on SBP and DBP.

The ratings regarding NutriGrade and AMSTAR 2 referred to all four SRs and, with one exception, achieved a rating of low regarding outcome-specific certainty of evidence and a split between low and moderate regarding methodological quality (Table 1D). There was some overlap of studies being used in the SRs (Supplementary Material S2G).

### Grading of the overall certainty of the evidence

Twelve SRs were used to grade the evidence of whether total protein and the subtypes animal and plant protein affect BP (Table 2). Four SRs [35, 36, 38, 39] were excluded from the evidence grading as they examined specifically milk and soy proteins.

**Table 2:** Overview of the systematic reviews used to grade the overall certainty of the evidence for the link between blood pressure and total, animal and plant protein

	Systematic review	Included study type	Protein and BP	NutriGrade rating	AMSTAR 2 rating
*(A) Total protein*	Hengeveld et al. [28]	RCTs	Ø	SBP: low DBP: low	Moderate
	Vogtschmidt et al. [27]	RCTs	↓: SBP Ø: DBP	SBP: low DBP: low	High
	Lonnie et al. [26]	RCTs	Ø	SBP: very low DBP: very low	Low
	Mousavi et al. [25]: high vs low analysis	Cohorts	Ø	Hypertension: moderate	Moderate
	Clifton et al. [24]	RCTs	Ø	SBP: low DBP: low	Moderate
	Pedersen et al. [22]	Cohorts and RCTs	Ø	SBP: low DBP: low Hypertension: moderate	Moderate
	Schwingshackl and Hoffmann [23]	RCTs	Ø	SBP: low DBP: low	High
	Wycherley et al. [21]	RCTs	Ø	SBP: low DBP: low	Low
	Rebholz et al. [19]	RCTs	↓	SBP: moderate DBP: moderate	Low
	Santesso et al. [20]: analysis with end of study values	RCTs	Ø	SBP: low DBP: low	High
	∑	*n* = 10 SRs on total protein: 9 Ø, 2 ↓ Majority of SRs reach at least a “low” NutriGrade rating Majority of SRs reach at least a “moderate” AMSTAR 2 rating			

	Systematic review	Included study type	Protein and BP	NutriGrade rating	AMSTAR 2 rating
*(B) Animal protein*	Mousavi et al. [25]: high vs low analysis	Cohorts	Ø	Hypertension: moderate	Moderate
	Chalvon-Demersay et al. [37]	Cohorts	Ø	SBP: low DBP: low	Moderate
	Pedersen et al. [22]	Cohorts and RCTs	Ø	SBP: low DBP: low Hypertension: moderate	Moderate
	Rebholz et al. [19]	RCTs	↓	SBP: moderate DBP: moderate	Low
	∑	*n* = 4 SRs on animal protein: 3 Ø, 1 ↓ Majority of SRs reach at least a “low” NutriGrade rating Majority of SRs reach at least a “moderate” AMSTAR 2 rating			

	Systematic review	Included study type	Protein and BP	NutriGrade rating	AMSTAR 2 rating
*(C) Plant protein*	Mousavi et al. [25]: high vs low analysis	Cohorts	Ø	Hypertension: moderate	Moderate
	Chalvon-Demersay et al. [37]	Cohorts	↓	SBP: moderate DBP: moderate	Moderate
	Pedersen et al. [22]	Cohorts	↓	SBP: low DBP: low Hypertension: high	Moderate
	Rebholz et al. [19]	RCTs	↓	SBP: moderate DBP: moderate	Low
	∑	*n* = 4 SRs on plant protein: 1 Ø, 3 ↓ Majority of SRs reach at least a “moderate” NutriGrade rating Majority of SRs reach at least a “moderate” AMSTAR 2 rating			

	Systematic review	Included study type	Protein and BP	NutriGrade rating	AMSTAR 2 rating
*(D) Plant vs animal protein*	Bryant et al. [40]	RCTs	Ø	SBP: low DBP: low	Moderate
	Lonnie et al. [26]	RCTs	Ø	SBP: low DBP: low	Moderate
	Chalvon-Demersay et al. [37]	RCTs	Ø	Moderate	Moderate
	Rebholz et al. [19]	RCTs	Ø	SBP: low DBP: low	Low
	∑	*n* = 4 SRs on plant vs animal protein: 4 Ø Majority of SRs reach at least a “low” NutriGrade rating Majority of SRs reach at least a “moderate” AMSTAR 2 rating			

*AMSTAR 2* A Measurement Tool to Assess Systematic Reviews 2, *BP* blood pressure, *DBP* diastolic blood pressure, *RCT* randomised controlled trial, *SBP* systolic blood pressure, *SR* systematic review, *Ø* no effect/association or inconsistent results, *↓* blood pressure decrease

Most of the ten SRs on total protein and BP reached at least a moderate methodological quality, a low rating of the outcome-specific certainty of evidence, and found no effect on BP (Table 2). Thus, the overall certainty of evidence regarding the BP-modulating influence of total protein was rated “possible” for having no effect on BP.

The four SRs on animal protein were mostly of moderate methodological quality and outcome-specific certainty of evidence, and the vast majority of the SRs found no effect of animal protein on BP (Table 2B). An exception was the SR by Rebholz et al. [19], which analysed a specific research question, namely, the replacement of carbohydrates with total protein or animal and plant protein. They found that such replacement reduced BP at all instances (total protein, animal and plant protein) (Table 2A–C). Overall, we concluded for animal protein that there is “possible” overall certainty of evidence for no effect.

Concerning the link between plant protein and BP, the majority of SRs analysing cohort studies showed an inverse association between plant protein intake and BP (Table 2C). However, this finding was counterbalanced by the four SRs of RCTs showing no relationship when comparing animal and plant protein directly, and was given greater weight for the assessment of the overall certainty of evidence than the SRs of cohort studies. Therefore, we concluded that there is no relation. Most of the SRs received moderate ratings in terms of methodological quality and outcome-specific certainty of evidence. Consequently, we graded the causal link between plant protein and BP as “possible” for no effect.

## Discussion

This umbrella review systematically evaluated the evidence on the role of dietary protein on BP or hypertension, yielding “possible” evidence for no link between total protein, animal protein and plant protein intake and BP. However, SRs which analysed exclusively special types of proteins such as milk or soy proteins were not included in the grading of the overall certainty of evidence. These proteins may have effects on BP that are different from those of total, animal or plant proteins in general. The grading of evidence regarding the role of dietary protein on BP in the current umbrella review is in line with findings of a recently published umbrella review on the role of diet in the prevention and management of hypertension [7]. This umbrella review indicated that the evidence for dietary protein overall, as well as for animal or vegetable protein, is of low quality.

The grading of the overall certainty of evidence depends not only on the conduct of the SRs but also on the availability and quality of the original studies. Both aspects are critical with respect to dietary protein and BP. Out of the 16 eligible SRs, six did not perform a formal MA [22, 26, 28, 37, 39, 40], and two SRs utilised only a subset of studies (see method section, Supplementary Material S7). The vast majority of SRs were rated “low” in terms of their outcome-specific certainty of evidence, despite the predominantly moderate or high methodological quality of most SRs. The reasons for the low outcome-specific certainty of evidence grading in most SRs resulted from the low number of included primary studies, which can cause publication bias and/or heterogeneity, and the existence of a potential conflict of interest (Supplementary Materials S7 and S8). In terms of the quality of original studies, we noted that many existing dietary cohort studies that addressed associations between diets and pathologies, evaluated data regarding BP change or incidence of hypertension, but failed to address specifically the association between protein intake and BP. BP is difficult to analyse in an observational setting due to the many factors affecting BP, and it is challenging to define the clinical diagnosis of primary hypertension because of the widespread use of antihypertensive medication and the presence of other diseases. In addition, some cohort studies have failed to consider important confounding factors for BP, such as other dietary factors associated with protein consumption. The critical remarks on the conduct of observational studies can also be applied to RCTs. Many RCTs did not include information on the use and type of medication or did not consider the effects of antihypertensive drugs. For example, in the SR of Rebholz et al. only 15 of 32 RCTs included subjects without BP-lowering medication [19]. Another factor that has a strong effect on BP is weight reduction. Many SRs included weight loss studies, although weight reduction is known to lower BP [41–43] and could dominate the presumed protein effect on BP.

In addition, another limitation is that the majority of studies included in the SRs used self-reports on dietary protein intake, although protein intake can be determined more precisely by renal nitrogen excretion [44, 45]. In view of the potential biases associated with self-reports of dietary intake, more studies using biomarkers or controlled protein applications are highly warranted. The latter aspect is important because many clinical trials studies used foods rich in protein and not protein isolates to address the protein effect on BP. Many trials advised the study participants to consume a diet high in protein from meat, fish, eggs or other animal sources (often referred to as an omnivorous diet), while the control diets were often more in line with a vegetarian diet. An umbrella review of MA of interventional and observational studies shows that individual food groups and dietary patterns can influence BP very differently due to their ingredients such as sodium, potassium, magnesium, plant compounds and fatty acids [7]. Thus, the use of proteins from food sources as an intervention measure instead of purified protein isolates could compromise the findings by providing additional bioactive compounds affecting BP, for example, isoflavones with soy protein. While such a phenomenon of confounding effects could in principle be addressed in RCTs, the seriousness of such bias is much greater in observational studies. In the past, information on bioactive compounds was not available in nutrient tables and thus could not be considered in the statistical analyses. Observational studies, particularly those addressing plant proteins without considering the potential effects of a high vegetable and fruit intake on BP [46] and for BP-modulating dietary bioactive compounds, have a high chance of confounding bias if showing an inverse association. In the grading of the evidence of an effect of plant protein on BP, we have thus weighted the RTCs comparing animal and plant proteins higher than findings of an inverse association in cohorts. Further, we excluded SRs from our evaluation of the evidence that addressed proteins from specific food sources.

In addition, RCTs typically involve a treatment group receiving additional dietary protein which is replaced totally or in part in the control group by similar quantities or energy-adjusted amounts of non-protein macronutrients such as carbohydrates or fat, or by providing different types of proteins in the intervention and control groups. Most studies investigated the effect of increasing dietary protein intake in the range of > 25 *E*%, but not the effect of reducing dietary protein intake from the current range of 15–20 *E*% to 10–12 *E* %, reflecting the protein requirement. Moreover, the replacement of other macronutrients by protein as being specifically analysed by Rebholz et al. [19] raises the question of whether the increasing protein intake or reducing, e.g. carbohydrates, causes the effect.

In contrast to total protein, animal protein and plant protein, proteins from specific food sources such as milk proteins are described to be efficient in BP reduction. Interestingly, SRs exclusively focussing on milk proteins, such as whey protein or casein hydrolysate, demonstrate favourable effects on BP [47, 48]. This is in line with a recently published umbrella review that found moderate quality evidence for a BP-lowering effect of lactotripeptides and a lower prevalence of hypertension associated with low-fat dairy, milk and fermented dairy consumption [7]. These results are in accordance with mechanistic data that identified specific peptides to be capable of modulating BP. A recent MA of 12 RCTs on food–protein–derived peptides found pooled effects of peptide intervention on SBP and DBP to be − 3.28 mm Hg and − 1.82 mm Hg, respectively [49]. Most peptides used in this MA were derived from milk and milk products, such as the casein-derived tripeptides valine–proline–proline and isoleucine–proline–proline, whey-derived lactokinin and fragments of α- and β-lactoglobulin which can inhibit ACE, thereby reducing the synthesis of angiotensin II and vasoconstriction [50–52]. These findings emphasise the role of specific peptides in lowering BP, and the need for more studies addressing peptides, rather than proteins in total.

The strengths of this umbrella review are (1) the standardised methodical procedures to include the entire appropriate literature, (2) the systematic literature search in three literature databases that aimed to include all relevant SRs and (3) the evaluation of the methodological quality as well as the evaluation of the outcome-specific certainty of evidence of the included SRs.

A weakness of this umbrella review is that the overall certainty of evidence is mainly based on data of SRs that included very heterogeneous RCTs in terms of the study population, study design, protein intake and control interventions, while methodically well-conducted SRs of observational studies are under-represented. It is crucial to note that the RCTs included in the SRs, which may not have specifically measured BP as the primary outcome, pose the risk of being underpowered to detect BP-related effects. This circumstance is also apparent in the assessment of the outcome-specific certainty of the evidence. In fact, about half of the outcome-specific NutriGrade assessments received a score of 0 points for the precision domain, due to low sample size (e.g. < 400 participants for a meta-analysis of RCTs) and/or wide 95% CIs, indicating a potential power issue. Furthermore, the best tool for rating the outcome-specific certainty of evidence warrants discussion. In our umbrella review, we chose NutriGrade, specifically developed to address the unique requirements of nutrition research [53]. Notably, the GRADE approach has undergone subsequent amendments and may emerge as the primary tool in the future. Generally, by considering all SRs from the last 10 years as commonly done in umbrella reviews earlier published studies are over-represented. We addressed this issue in Supplementary Material S2, where the original study overlap is explored. The analysis proved insightful, revealing that there was only a moderate overlap among the SRs regarding the primary studies. Finally, to mitigate the risk of overlooking relevant SRs published recently, an updated literature search was conducted in PubMed in November 2023, using our original search strategy. The search identified two additional SRs, of which one included only three RCTs, which were already considered in other SRs included in our umbrella review [54]. The second SR, specifically addressing milk proteins, is discussed above [47]. Importantly, the findings from these additional SRs do not alter the key messages of our umbrella review.

## Conclusion

This umbrella review showed uncertainties regarding the link between BP and the intake of total protein, as well as animal or plant proteins specifically. The methodological quality of the SRs ranged from low to high, and the outcome-specific certainty of evidence was mostly low. Future high-quality RCTs using well-characterised study populations, defined quantities of proteins or valid assessments of protein intake and iso-energetic control interventions are warranted to provide high-quality evidence and a solid basis for recommendations on dietary protein and BP.

### Supplementary Information

Below is the link to the electronic supplementary material.

Supplementary file1 (DOCX 16 KB)

Supplementary file2 (DOCX 47 KB)

Supplementary file3 (DOCX 32 KB)

Supplementary file4 (DOCX 63 KB)

Supplementary file5 (DOCX 19 KB)

Supplementary file6 (DOCX 30 KB)

Supplementary file7 (DOCX 23 KB)

Supplementary file8 (DOCX 37 KB)

Supplementary file9 (XLSX 16 KB)

## Data Availability

Not applicable.

## Abbreviations

AMSTAR 2: A Measurement Tool to Assess Systematic Reviews 2
AS: Amino acids
BP: Blood pressure
CI: Confidence interval
CVD: Cardiovascular diseases
DASH: Dietary approaches to stop hypertension
DBP: Diastolic blood pressure
MAs: Meta-analysis/meta-analyses
RCT: Randomised controlled trial
SBP: Systolic blood pressure
SR: Systematic review
