# Supplementary material for: Dietary protein and blood pressure: an umbrella review of systematic reviews and evaluation of the evidence
Source: Eur J Nutr. 2024 Feb 20;63(4):1041–58. doi: 10.1007/s00394-024-03336-8 (PMC11139777; doi:10.1007/s00394-024-03336-8)
Supplement: Supplementary file 2 — Supplementary file2 (DOCX 47 KB) [file 394_2024_3336_MOESM2_ESM.docx]

**Supplementary Material S2.** Overview of primary studies being analysed in the SRs.

1. **Total protein studies: List of included RCTs**

| **Systematic review**  **Included RCTs** | | **Rebholz**  **2012** | **Santesso**  **2012** | **Wycherley**  **2012** | **Pedersen**  **2013** | **Schwingshackl**  **2013** | **Clifton**  **2014** | **Lonnie**  **2020** | **Vogtschmidt**  **2021** | **Hengeveld**  **2022** |
| --- | --- | --- | --- | --- | --- | --- | --- | --- | --- | --- |
| Included in 5 SRs | Delbridge 2009 | X | x |  |  | x | x |  | x |  |
| Included in 4 SRs | Appel 2005 | X | x |  | X |  |  |  | x |  |
|  | Dansinger 2005 |  | x |  |  | x | x |  | x |  |
|  | McAuley 2005 | x | x |  |  | x | x |  |  |  |
|  | Gardner 2007 |  | x |  |  | x | x |  | x |  |
|  | Leidy 2007 | x | x | x |  |  |  |  | x |  |
|  | Wycherley 2012 |  |  | x |  | x | x |  | x |  |
| Included in 3 SRs | Jenkins 2001 | x | x |  |  |  |  |  | x |  |
|  | Brinkworth 2004a | x |  |  |  | x | x |  |  |  |
|  | Brinkworth 2004b | x |  |  |  | x | x |  |  |  |
|  | Stamets 2004 |  | x | x |  |  |  |  | x |  |
|  | Ferrara 2006 | x | x |  |  |  |  |  | x |  |
|  | Hodgson 2006 | x | x |  |  |  |  |  | x |  |
|  | Keogh 2007b |  |  |  |  | x | x |  | x |  |
|  | Abete 2009 | x | x |  |  |  |  |  | x |  |
| Included in 2 SRs | Yancy 2004 | x | x |  |  |  |  |  |  |  |
|  | Brehm 2005 | x | x |  |  |  |  |  |  |  |
|  | Kleiner 2006 |  | x |  |  |  |  |  | x |  |
|  | Keogh 2007a |  | x |  |  |  | x |  |  |  |
|  | Maki 2007 |  | x |  |  |  |  |  | x |  |
|  | Meckling 2007 | x |  |  |  |  |  |  | x |  |
|  | Brinkworth 2009 |  | x |  |  |  | x |  |  |  |
|  | De Luis 2009 |  | x |  |  |  |  |  | x |  |
|  | Sacks 2009 |  |  |  |  | x | x |  |  |  |
|  | Krebs 2010 |  |  |  |  | x | x |  |  |  |
|  | Aldrich 2011 |  | x |  |  |  |  |  | x |  |
|  | Larsen 2011 |  |  |  |  | x | x |  |  |  |
|  | Te Morenga 2011 |  | x |  |  |  |  |  | x |  |
|  | Wright 2018 |  |  |  |  |  |  |  | x | x |
| Included in 1 SR | Sacks 1984 |  | x |  |  |  |  |  |  |  |
|  | Rouse 1986 |  | x |  |  |  |  |  |  |  |
|  | DeHaven 1988 | x |  |  |  |  |  |  |  |  |
|  | Hendler 1988 | x |  |  |  |  |  |  |  |  |
|  | Lean 1997 |  | x |  |  |  |  |  |  |  |
|  | Washburn 1999 | x |  |  |  |  |  |  |  |  |
|  | Burke 2001 | x |  |  |  |  |  |  |  |  |
|  | Teede 2001 | x |  |  |  |  |  |  |  |  |
|  | Parker 2002 |  |  | x |  |  |  |  |  |  |
|  | Brehm 2003 |  | x |  |  |  |  |  |  |  |
|  | Foster 2003 |  | x |  |  |  |  |  |  |  |
|  | Samaha 2003 | x |  |  |  |  |  |  |  |  |
|  | Due 2004 |  | x |  |  |  |  |  |  |  |
|  | Harrison 2004 | x |  |  |  |  |  |  |  |  |
|  | Sagara 2004 | x |  |  |  |  |  |  |  |  |
|  | Stern 2004 |  |  |  |  |  | x |  |  |  |
|  | He 2005 | x |  |  |  |  |  |  |  |  |
|  | Muzio 2007 |  | x |  |  |  |  |  |  |  |
|  | Dipla 2008 | x |  |  |  |  |  |  |  |  |
|  | Morgan 2008 |  | x |  |  |  |  |  |  |  |
|  | Shai 2008 |  |  |  |  |  | x |  |  |  |
|  | Tay 2008 | x |  |  |  |  |  |  |  |  |
|  | Al-Sarraj 2009 | x |  |  |  |  |  |  |  |  |
|  | Buscemi 2009 |  | x |  |  |  |  |  |  |  |
|  | Frisch 2009 |  |  |  |  |  | x |  |  |  |
|  | Jenkins 2009 |  | x |  |  |  |  |  |  |  |
|  | Pal 2009 | x |  |  |  |  |  |  |  |  |
|  | Belobrajdic 2010 |  |  | x |  |  |  |  |  |  |
|  | Klemsdal 2010 |  |  |  |  |  | x |  |  |  |
|  | Wycherley 2010 | x |  |  |  |  |  |  |  |  |
|  | Gögebakan 2011 |  |  |  |  |  |  |  | x |  |
|  | He 2011 | x |  |  |  |  |  |  |  |  |
|  | van Meijl 2011 | x |  |  |  |  |  |  |  |  |
|  | Gulbrand 2012 |  |  |  |  |  | x |  |  |  |
|  | Hodgson 2012 |  |  |  |  |  |  |  |  | x |
|  | Soenen 2012 |  |  |  |  |  | x |  |  |  |
|  | Azadbakht 2013 |  |  |  |  |  |  |  | x |  |
|  | Tang 2013 |  |  |  |  |  |  |  | x |  |
|  | Jenkins 2014 |  |  |  |  |  |  |  | x |  |
|  | Teunissen-Beekman 2014 |  |  |  |  |  |  | x |  |  |
|  | Mateo-Gallego 2017 |  |  |  |  |  |  |  | x |  |
|  | Nabuco 2018 |  |  |  |  |  |  |  |  | x |
|  | Nabuco 2019 |  |  |  |  |  |  |  |  | x |
|  | Gonzalez-Salazar 2020 |  |  |  |  |  |  |  | x |  |

1. **Total protein studies: List of included cohort studies**

| **Systematic review**  **Included cohort studies** | | **Pedersen**  **2013** | **Mousavi**  **2020** |
| --- | --- | --- | --- |
| Included  in 2 SRs | Alonso 2006 | x | x |
| Included in 1 SR | Stamler 2002 | x |  |
|  | Altorf-van der Kuil 2010 |  | x |
|  | Altorf-van der Kuil 2012 |  | x |
|  | Buenida 2015 |  | x |
|  | Lelong 2017 |  | x |

1. **Animal protein studies: List of included RCTs**

| **Systematic review**  **Included RCTs** | | **Rebholz**  **2012** | **Hidayat**  **2017** | **Badely**  **2019** |
| --- | --- | --- | --- | --- |
| Included in 3 SRs | Pal 2009 | x | x | x |
| Included in 2 SRs | Petyaev 2012 |  | x | x |
|  | Vatani 2012 |  | x | x |
|  | Figueroa 2013 |  | x | x |
| Included in 1 SR | DeHaven 1988 | x |  |  |
|  | Hendler 1988 | x |  |  |
|  | Brinkworth 2004 | x |  |  |
|  | Ferrara 2006 | x |  |  |
|  | Hodgson 2006 | x |  |  |
|  | Pins 2006 |  |  | x |
|  | Leidy 2007 | x |  |  |
|  | Meckling 2007 | x |  |  |
|  | Dipla 2008 | x |  |  |
|  | Abete 2009 | x |  |  |
|  | Al-Sarraj 2009 | x |  |  |
|  | Daly 2009 |  | x |  |
|  | Delbridge 2009 | x |  |  |
|  | Fluegel 2010 |  |  | x |
|  | Aldrich 2011 |  |  | x |
|  | He 2011 | x |  |  |
|  | Hodgson 2011 |  |  | x |
|  | van Meijl 2011 | x |  |  |
|  | Petrogianni 2012 |  | x |  |
|  | Beavers 2015 |  |  | x |
|  | Tahavorgar 2015 |  |  | x |
|  | Tovar 2015 |  |  | x |
|  | Arciero 2016 |  |  | x |
|  | Fekete 2016 |  |  | x |
|  | Lee 2016 |  | x |  |
|  | Gulati 2017 |  |  | x |
|  | Larsen 2018 |  |  | x |
|  | Mohammadi-S. 2018 |  |  | x |
|  | Hellen 2019 |  |  | x |
|  | Kjolbaek 2019 |  |  | x |

1. **Animal protein studies: List of included cohort studies**

| **Systematic review**  **Included RCTs** | | **Pedersen**  **2013** | **Chalvon-Demersay 2017** | **Mousavi**  **2020** |
| --- | --- | --- | --- | --- |
| Included in 2 SRs | Stamler 2002 | x | x |  |
|  | Alonso 2006 | x |  | x |
| Included in 1 SR | Wang 2008 |  | x |  |
|  | Altorf-van der Kuil 2010 |  |  | x |
|  | Altorf-van der Kuil 2012 |  |  | x |
|  | Tielemans 2014 |  | x |  |
|  | Buenida 2015 |  | x |  |
|  | Lelong 2017 |  |  | x |

1. **Plant protein studies: List of included RCTs**

| **Systematic review**  **Included RCTs** | | **Rebholz**  **2012** | **Mohammadifard**  **2021** | **Mosallanezhad**  **2021** |
| --- | --- | --- | --- | --- |
| Included in 2 SRs | Teede 2001 | x |  | x |
|  | Sagara 2004 | x |  | x |
|  | He 2005 | x |  | x |
| Included in 1 SR | Washburn 1999 | x |  |  |
|  | Burke 2001 | x |  |  |
|  | Jenkins 2001 | x |  |  |
|  | Harrison 2004 | x |  |  |
|  | Hermansen 2005 |  |  | x |
|  | Azadbakht 2007 |  | x |  |
|  | He 2011 | x |  |  |
|  | Bakhtiari 2019 |  | x |  |

1. **Plant protein studies: List of included cohort studies**

| **Systematic review**  **Included cohort studies** | | **Pedersen**  **2013** | **Chalvon-Demersay 2017** | **Mousavi**  **2020** |
| --- | --- | --- | --- | --- |
| Included in 2 SRs | Stamler 2002 | x | x |  |
|  | Alonso 2006 | x |  | x |
| Included in 1 SR | Wang 2008 |  | x |  |
|  | Altorf-van der Kuil 2010 |  |  | x |
|  | Altorf-van der Kuil 2012 |  |  | x |
|  | Tielemans 2014 |  | x |  |
|  | Buenida 2015 |  | x |  |
|  | Lelong 2017 |  |  | x |

1. **Animal vs plant protein studies overlap of included RCTs**

| **Systematic review**  **Included RCTs** | | **Rebholz**  **2012** | **Chalvon-Demersay**  **2017** | **Lonnie**  **2020** | **Bryant**  **2022** |
| --- | --- | --- | --- | --- | --- |
| Included in 3 SRs | Bähr 2013 |  | x | x | x |
| Included in 2 SRs | Brussaard 1981 | x | x |  |  |
|  | Teede 2001 | x | x |  |  |
|  | Cuevas 2003 | x | x |  |  |
|  | He 2011 | x | x |  |  |
| Included in 1 SR | Margetts 1985 | x |  |  |  |
|  | Prescott 1988 | x |  |  |  |
|  | Vigna 2000 | x |  |  |  |
|  | Hermansen 2001 | x |  |  |  |
|  | Jenkins 2001 | x |  |  |  |
|  | Wheeler 2002 | x |  |  |  |
|  | Meyer 2004 | x |  |  |  |
|  | Anderson 2005 |  | x |  |  |
|  | Kreijkamp-Kaspers 2005 |  | x |  |  |
|  | Matthan 2007 | x |  |  |  |
|  | Jenkins 2010 |  | x |  |  |
|  | Liu 2012 |  | x |  |  |
|  | Bähr 2014 |  |  | x |  |
|  | Bähr 2015 |  |  |  | x |
|  | Beavers 2015 |  | x |  |  |
