# Supplementary material for: Dietary protein and blood pressure: an umbrella review of systematic reviews and evaluation of the evidence
Source: Eur J Nutr. 2024 Feb 20;63(4):1041–58. doi: 10.1007/s00394-024-03336-8 (PMC11139777; doi:10.1007/s00394-024-03336-8)
Supplement: Supplementary file 5 — Supplementary file5 (DOCX 19 KB) [file 394_2024_3336_MOESM5_ESM.docx]

**Supplementary Material S5.** Grading the overall certainty of evidence according to methodological quality, outcome-specific certainty of evidence, biological plausibility and consistency of results, and definition of the overall certainty of evidence in a modified form according to the GRADE approach [1, 2].

| Overall certainty of evidence | Underlying criteria | Definition/Explanation |
| --- | --- | --- |
| Convincing | - At least one SR with or without MA of prospective studies available - If more than one SR with or without MA are available: all overall results must be consistent.^1^ - In case of a positive or negative association, biological plausibility is given - All included SRs with or without MA must reach at least a “moderate” outcome-specific certainty of evidence^2^; in addition all included SRs must reach at least a methodological quality^3^ of “moderate” | There is high level of confidence that the true effect lies close to that of the estimate(s) of the effect |
| Probable | - At least one SR with or without MA of prospective studies available - If more than one SR with or without MA are available, the majority of overall results must be consistent.^1^ - In case of a positive or negative association, biological plausibility is given - The majority^4^ of included SRs with or without MA must have reached at least a “moderate” outcome-specific certainty of evidence^2^; in addition all included SRs must reach at least a methodological quality^3^ of “moderate” | There is moderate confidence in the effect estimate(s):  The true effect is likely to be close to the estimate of the effect, but there is a possibility that it is substantially different |
| Possible | - At least one SR with or without MA of prospective studies available - If more than one SR with or without MA are available, the majority of overall results must be consistent.^1^ - In case of a positive or negative association, biological plausibility is given - The majority^4^ of included SRs with or without MA must reach at least a “low” outcome-specific certainty of evidence^2^; in addition the majority^4^ of all included SRs must reach at least a methodological quality^3^ of “moderate” | Confidence in the effect estimate(s) is limited:  The true effect may be substantially different from the estimate of the effect |
| Insufficient | - No SR is available   *OR*   - The majority^4^ of included SRs with or without MA reach a “very low” outcome-specific certainty of evidence^2^; in addition the majority of all included SRs reach a methodological quality^3^ of “low” | There is very little confidence in the effect estimate (s):  The true effect is likely to be substantially different from the estimate of effect |

^1^ Consistent = overall results of the SR have to be consistently either risk reducing or risk elevating or consistently showing no risk association

^2^ Outcome-specific certainty of evidence refers to the NutriGrade rating

^3^ Methodological quality refers the AMSTAR 2 rating**;** SRs graded as “critically low” by AMSTAR 2 are not considered.

^4^ Majority: > 50 % of the included SRs

Abbreviations: MA: meta-analysis; SR: systematic review

**References**

1 Alonso-Coello P, Schünemann HJ, Moberg J et al. (2016) GRADE Evidence to Decision (EtD) frameworks: a systematic and transparent approach to making well informed healthcare choices. 1: Introduction. BMJ 353:i2016. doi: 10.1136/bmj.i2016

2 Kroke A, Schmidt A, Amini AM, Kalotai N, Lehmann A, Bauer JM, Bischoff-Ferrari HA, Boeing H, Egert S, Ellinger S, Kühn T, Louis S, Lorkowski S, Nimptsch K, Remer T, Schulze MB, Siener R, Stangl GI, Volkert D, Zittermann A, Buykens AE, Watzl B, Schwingshackl L: Dietary protein intake and health-related outcomes: a methodological approach for the evidence-based guideline of the German Nutrition Society. Eur J Nutr 2022 doi:10.1007/s00394-021-02789-5
