# Supplementary material for: Dietary protein and blood pressure: an umbrella review of systematic reviews and evaluation of the evidence
Source: Eur J Nutr. 2024 Feb 20;63(4):1041–58. doi: 10.1007/s00394-024-03336-8 (PMC11139777; doi:10.1007/s00394-024-03336-8)
Supplement: Supplementary file 6 — Supplementary file6 (DOCX 30 KB) [file 394_2024_3336_MOESM6_ESM.docx]

Supplemental Material S6. List of excluded articles and the reasons.

| **Reference** | **Reason for exclusion** |
| --- | --- |
| Abbasnezhad A, Falahi E, Gonzalez MJ et al. (2020) Effect of different dietary approaches compared with a regular diet on systolic and diastolic blood pressure in patients with type 2 diabetes: A systematic review and meta-analysis. Diabetes Res Clin Pract 163:108108. | Irrelevant population |
| Abedini M, Falahi E, Roosta S (2015) Dairy product consumption and the metabolic syndrome. Diabetes Metab Syndr 9:34–37. | Relevant diet-disease relationship not investigated |
| Afolayan AJ, Wintola OA (2014) Dietary supplements in the management of hypertension and diabetes - a review. Afr J Tradit Complement Altern Med 11:248–258. | Relevant diet-disease relationship not investigated |
| Ali Redha A, Valizadenia H, Siddiqui SA et al. (2022) A state-of-art review on camel milk proteins as an emerging source of bioactive peptides with diverse nutraceutical properties. Food Chem 373:131444. | Irrelevant exposure |
| Aljefree N, Ahmed F (2015) Association between dietary pattern and risk of cardiovascular disease among adults in the Middle East and North Africa region: a systematic review. Food Nutr Res 59:27486. | Relevant diet-disease relationship not investigated |
| Altorf-van der Kuil W, Engberink MF, Brink EJ et al. (2010) Dietary protein and blood pressure: a systematic review. PLoS One 5:e12102. | Critically low AMSTAR 2 rating |
| Amirani E, Milajerdi A, Reiner Ž et al. (2020) Effects of whey protein on glycemic control and serum lipoproteins in patients with metabolic syndrome and related conditions: a systematic review and meta-analysis of randomized controlled clinical trials. Lipids Health Dis 19:209. | Irrelevant outcome |
| Anne Fernandez M, Picard-Deland É, Daniel N et al. (2017) Yogurt and health: overview of recent data. Cahiers de Nutrition et de Diététique 52, Suppl:S48-S57. | Irrelevant language |
| Arabi SM, Bahrami LS, Milkarizi N et al. (2022) Impact of walnut consumption on cardio metabolic and anthropometric parameters in metabolic syndrome patients: GRADE-assessed systematic review and dose-response meta-analysis of data from randomized controlled trials. Pharmacol Res 178:106190. | Irrelevant exposure |
| Asbaghi O, Hadi A, Campbell MS et al. (2021) Effects of pistachios on anthropometric indices, inflammatory markers, endothelial function and blood pressure in adults: a systematic review and meta-analysis of randomised controlled trials. Br J Nutr 126:718–729. | Irrelevant exposure |
| Battaglia Richi E, Baumer B, Conrad B et al. (2015) Health risks associated with meat consumption: a review of epidemiological studies. Int J Vitam Nutr Res 85:70–78. | Irrelevant outcome |
| Bazzano LA, Green T, Harrison TN et al. (2013) Dietary approaches to prevent hypertension. Curr Hypertens Rep 15:694–702. | Irrelevant study type |
| Benatar JR, Sidhu K, Stewart RAH (2013) Effects of high and low fat dairy food on cardio-metabolic risk factors: a meta-analysis of randomized studies. PLoS One 8:e76480. | Relevant diet-disease relationship not investigated |
| Bito T, Okumura E, Fujishima M et al. (2020) Potential of chlorella as a dietary supplement to promote human health. Nutrients 12:2524. | Irrelevant exposure |
| Blanco Mejia S, Kendall CWC, Viguiliouk E et al. (2014) Effect of tree nuts on metabolic syndrome criteria: a systematic review and meta-analysis of randomised controlled trials. BMJ Open 4:e004660. | Relevant diet-disease relationship not investigated |
| Boelsma E, Kloek J (2009) Lactotripeptides and antihypertensive effects: a critical review. Br J Nutr 101:776–786. | Irrelevant exposure |
| Bonnet JP, Cardel MI, Cellini J et al. (2020) Breakfast skipping, body composition, and cardiometabolic risk: a systematic review and meta-analysis of randomized trials. Obesity (Silver Spring) 28:1098–1109. | Irrelevant exposure |
| Borghi C, Cicero AFG (2017) Nutraceuticals with a clinically detectable blood pressure-lowering effect: a review of available randomized clinical trials and their meta-analyses. Br J Clin Pharmacol 83:163–171. | Irrelevant study type |
| Buendia JR, Li Y, Hu FB et al. (2018) Long-term yogurt consumption and risk of incident hypertension in adults. J Hypertens 36:1671–1679. | Relevant diet-disease relationship not investigated |
| Castro I, Waclawovsky G, Marcadenti A (2015) Nutrition and physical activity on hypertension: implication of current evidence and guidelines. Curr Hypertens Rev 11:91–99. | Irrelevant study type |
| Chanson-Rolle A, Aubin F, Braesco V et al. (2015) Influence of the lactotripeptides isoleucine-proline-proline and valine-proline-proline on systolic blood pressure in japanese subjects: a systematic review and meta-analysis of randomized controlled trials. PLoS One 10:e0142235. | Irrelevant exposure |
| Chen G-C, Szeto IMY, Chen L-H et al. (2015) Dairy products consumption and metabolic syndrome in adults: systematic review and meta-analysis of observational studies. Sci Rep 5:14606. | Irrelevant exposure |
| Chen Z, Ahmed M, Ha V et al. (2021) Dairy product consumption and cardiovascular health: a systematic review and meta-analysis of prospective cohort studies. Adv Nutr. | Irrelevant exposure |
| Chiavaroli L, Lee D, Ahmed A et al. (2021) Effect of low glycaemic index or load dietary patterns on glycaemic control and cardiometabolic risk factors in diabetes: systematic review and meta-analysis of randomised controlled trials. BMJ 374:n1651. | Irrelevant population |
| Choi JH, Cho YJ, Kim H-J et al. (2022) Effect of carbohydrate-restricted diets and intermittent fasting on obesity, type 2 diabetes mellitus, and hypertension management: consensus statement of the Korean Society for the Study of Obesity, Korean Diabetes Association, and Korean Society of Hypertension. Diabetes Metab J 46:355–376. | Irrelevant exposure |
| Choi YJ, Jeon S-M, Shin S (2020) Impact of a ketogenic diet on metabolic parameters in patients with obesity or overweight and with or without type 2 diabetes: a meta-analysis of randomized controlled trials. Nutrients 12:2005. | Irrelevant exposure |
| Chrysant SG (2016) The clinical significance and costs of herbs and food supplements used by complementary and alternative medicine for the treatment of cardiovascular diseases and hypertension. J Hum Hypertens 30:1–6. | Irrelevant study type |
| Cicero AFG, Aubin F, Azais-Braesco V et al. (2013) Do the lactotripeptides isoleucine-proline-proline and valine-proline-proline reduce systolic blood pressure in European subjects? A meta-analysis of randomized controlled trials. Am J Hypertens 26:442–449. | Irrelevant exposure |
| Cicero AFG, Colletti A (2015) Nutraceuticals and blood pressure control: results from clinical trials and meta-analyses. High Blood Press Cardiovasc Prev 22:203–213. | Irrelevant study type |
| Cicero AFG, Gerocarni B, Laghi L et al. (2011) Blood pressure lowering effect of lactotripeptides assumed as functional foods: a meta-analysis of current available clinical trials. J Hum Hypertens 25:425–436. | Irrelevant exposure |
| Colonetti T, Grande AJ, Milton K et al. (2017) Effects of whey protein supplement in the elderly submitted to resistance training: systematic review and meta-analysis. Int J Food Sci Nutr 68:257–264. | Irrelevant outcome |
| Companys J, Pla-Pagà L, Calderón-Pérez L et al. (2020) Fermented dairy products, probiotic supplementation, and cardiometabolic diseases: a systematic review and meta-analysis. Adv Nutr 11:834–863. | Irrelevant exposure |
| Crichton GE, Bryan J, Buckley J et al. (2011) Dairy consumption and metabolic syndrome: a systematic review of findings and methodological issues. Obes Rev 12:e190-201. | Relevant diet-disease relationship not investigated |
| Da Silva MS, Rudkowska I (2014) Dairy products on metabolic health: current research and clinical implications. Maturitas 77:221–228. | Irrelevant study type |
| Dahl IK, Dalgård C (2021) Sami dietary habits and the risk of cardiometabolic disease: a systematic review. Int J Circumpolar Health 80:1873621. | Irrelevant exposure |
| de Morais Cardoso L, Pinheiro SS, Martino HSD et al. (2017) Sorghum (Sorghum bicolor L.): Nutrients, bioactive compounds, and potential impact on human health. Crit Rev Food Sci Nutr 57:372–390. | Irrelevant study type |
| Del Gobbo LC, Falk MC, Feldman R et al. (2015) Effects of tree nuts on blood lipids, apolipoproteins, and blood pressure: systematic review, meta-analysis, and dose-response of 61 controlled intervention trials. Am J Clin Nutr 102:1347–1356. | Relevant diet-disease relationship not investigated |
| Ding M, Huang T, Bergholdt HK et al. (2017) Dairy consumption, systolic blood pressure, and risk of hypertension: Mendelian randomization study. BMJ 356:j1000. | Relevant diet-disease relationship not investigated |
| Dong J-Y, Qin L-Q, Zhang Z et al. (2011) Effect of oral L-arginine supplementation on blood pressure: a meta-analysis of randomized, double-blind, placebo-controlled trials. Am Heart J 162:959–965. | Relevant diet-disease relationship not investigated |
| Dong J-Y, Szeto IMY, Makinen K et al. (2013) Effect of probiotic fermented milk on blood pressure: a meta-analysis of randomised controlled trials. Br J Nutr 110:1188–1194. | Irrelevant exposure |
| Dong J-Y, Tong X, Wu Z-W et al. (2011) Effect of soya protein on blood pressure: a meta-analysis of randomised controlled trials. Br J Nutr 106:317–326. | Critically low AMSTAR 2 rating |
| Dreher ML (2021) A comprehensive review of almond clinical trials on weight measures, metabolic health biomarkers and outcomes, and the gut microbiota. Nutrients 13:1968. | Irrelevant exposure |
| Drouin-Chartier J-P, Brassard D, Tessier-Grenier M et al. (2016) Systematic review of the Association between Dairy Product Consumption and Risk of Cardiovascular-Related Clinical Outcomes. Adv Nutr 7:1026–1040. | Relevant diet-disease relationship not investigated |
| Duyuler S, Özbek K, Balci KG et al. (2020) Egg consumption and cardiovascular health: how many eggs a day keep the doctor away? Angiology 71:667. | Irrelevant study type |
| Dzuvor CKO, Pan S, Amanze C et al. (2022) Bioactive components from Moringa oleifera seeds: production, functionalities and applications - a critical review. Crit Rev Biotechnol 42:271–293. | Irrelevant exposure |
| Earl GL, Henstenburg JA (2012) Dietary approaches to hypertension: a call to pharmacists to promote lifestyle changes. J Am Pharm Assoc (2003) 52:637–645. | Relevant diet-disease relationship not investigated |
| Elwood PC, Givens DI, Beswick AD et al. (2008) The survival advantage of milk and dairy consumption: an overview of evidence from cohort studies of vascular diseases, diabetes and cancer. J Am Coll Nutr 27:723–734. | Relevant diet-disease relationship not investigated |
| Fekete AA, Givens DI, Lovegrove JA (2015) Casein-derived lactotripeptides reduce systolic and diastolic blood pressure in a meta-analysis of randomised clinical trials. Nutrients 7:659–681. | Irrelevant exposure |
| Feng Y, Zhao Y, Liu J et al. (2022) Consumption of dairy products and the risk of overweight or obesity, hypertension, and type 2 diabetes mellitus: a dose-response meta-analysis and systematic review of cohort studies. Adv Nutr. https://doi.org/10.1093/advances/nmac096 | Irrelevant exposure |
| Fernandez MA, Panahi S, Daniel N et al. (2017) Yogurt and Cardiometabolic Diseases: A Critical Review of Potential Mechanisms. Adv Nutr 15:812–829. | Irrelevant study type |
| Feskens EJM, Sluik D, van Woudenbergh GJ (2013) Meat consumption, diabetes, and its complications. Curr Diab Rep (Current diabetes reports) 13:298–306. | Relevant diet-disease relationship not investigated |
| Fontecha J, Visitación Calvo M, Juarez M et al. (2019) Milk and dairy product consumption and cardiovascular diseases: an overview of systematic reviews and meta-analyses. Adv Nutr 10, Suppl 2:S164-S189. | Relevant diet-disease relationship not investigated |
| Gao Q, Qin L-Q, Arafa A et al. (2020) Effects of strawberry intervention on cardiovascular risk factors: a meta-analysis of randomised controlled trials. Br J Nutr 124:241–246. | Irrelevant exposure |
| Garvey WT, Mechanick JI, Brett EM et al. (2016) American Association of Clinical Endocrinologists and American College of Endocrinology comprehensive clinicial practice guidelines for medical care of patients with obesity. Endocr Pract 22 Suppl 3:1–203. | Irrelevant population |
| Ge L, Sadeghirad B, Ball GDC et al. (2020) Comparison of dietary macronutrient patterns of 14 popular named dietary programmes for weight and cardiovascular risk factor reduction in adults: systematic review and network meta-analysis of randomised trials. BMJ 369:m696. | Irrelevant exposure |
| Geleijnse JM, Engberink MF (2010) Lactopeptides and human blood pressure. Curr Opin Lipidol 21:58–63. | Irrelevant study type |
| Giosuè A, Calabrese I, Vitale M et al. (2022) Consumption of dairy foods and cardiovascular disease: a systematic review. Nutrients 14. | Irrelevant exposure |
| Giugliano D, Ceriello A, Esposito K (2008) Are there specific treatments for the metabolic syndrome? Am J Clin Nutr 87:8–11. | Irrelevant study type |
| Gonzalez-Campoy JM, St Jeor ST, Castorino K et al. (2013) Clinical practice guidelines for healthy eating for the prevention and treatment of metabolic and endocrine diseases in adults: cosponsored by the American Association of Clinical Endocrinologists/the American College of Endocrinology and the Obesity Society. Endocr Pract 19 Suppl 3:1–82. | Irrelevant study type |
| Guasch-Ferré M, Satija A, Blondin SA et al. (2019) Meta-analysis of randomized controlled trials of red meat consumption in comparison with various comparison diets on cardiovascular risk factors. Circulation 139:1828–1845. | Irrelevant outcome |
| Hackam DG, Khan NA, Hemmelgarn BR et al. (2010) The 2010 Canadian Hypertension Education Program recommendations for the management of hypertension: part 2 - therapy. Can J Cardiol 26:249–258. | Relevant diet-disease relationship not investigated |
| Hajizadeh-Sharafabad F, Sharifi Zahabi E, Tarighat-Esfanjani A (2021) Role of whey protein in vascular function: a systematic review and meta-analysis of human intervention studies. Br J Nutr:1–14. | Irrelevant outcome |
| He S, Stein AD (2021) Early-Life nutrition interventions and associated long-term cardiometabolic outcomes: a systematic review and meta-analysis of randomized controlled trials. Adv Nutr 12:461–489. | Irrelevant exposure |
| Heidari Z, Rashidi Pour Fard N, Clark CCT et al. (2021) Dairy products consumption and the risk of hypertension in adults: an updated systematic review and dose-response meta-analysis of prospective cohort studies. Nutr Metab Cardiovasc Dis 31:1962–1975. | Irrelevant exposure |
| Hession M, Rolland C, Kulkarni U et al. (2009) Systematic review of randomized controlled trials of low-carbohydrate vs. low-fat/low-calorie diets in the management of obesity and its comorbidities. Obes Rev 10:36–50. | Irrelevant exposure |
| Horikawa C, Kodama S, Heianza Y et al. (2012) Relationship between Dairy Intake and Risk of Metabolic Syndrome: A Meta-Analysis. Diabetes 61, Suppl 1:A 514. | Relevant diet-disease relationship not investigated |
| Hu T, Mills KT, Yao L et al. (2012) Effects of low-carbohydrate diets versus low-fat diets on metabolic risk factors: a meta-analysis of randomized controlled clinical trials. Am J Epidemiol 176 Suppl 7:S44-S54. | Irrelevant exposure |
| Jamioł-Milc D, Biernawska J, Liput M et al. (2021) Seafood intake as a method of non-communicable diseases (NCD) prevention in adults. Nutrients 13:1422. | Irrelevant study type |
| Jayachandran M, Xu B (2019) An insight into the health benefits of fermented soy products. Food Chem 271:362–371. | Irrelevant study type |
| Jayawardena R, Sooriyaarachchi P, Misra A (2021) Abdominal obesity and metabolic syndrome in South Asians: prevention and management. Expert Rev Endocrinol Metab 16:339–349. | Irrelevant exposure |
| Jin S, Je Y (2021) Dairy consumption and risk of metabolic syndrome: results from Korean population and meta-analysis. Nutrients 13:1574. | Irrelevant exposure |
| Jun M, Xiang Y (2020) The management of prehypertension in young adults. Saudi Med J 41:223–231. | Irrelevant exposure |
| Jun S, Ha K, Chung S et al. (2016) Meat and milk intake in the rice-based Korean diet: impact on cancer and metabolic syndrome. Proc Nutr Soc 75:374–384. | Relevant diet-disease relationship not investigated |
| Kairey L, Leech B, El-Assaad F et al. (2022) The effects of kefir consumption on human health: a systematic review of randomized controlled trials. Nutr Rev. https://doi.org/10.1093/nutrit/nuac054 | Irrelevant exposure |
| Kalyoncu ZB, Pars H, Bora-Güneş N et al. (2014) A systematic review of nutrition-based practices in prevention of hypertension among healthy youth. Turk J Pediatr 56:335–346. | Relevant diet-disease relationship not investigated |
| Kanerva N, Kaartinen NE, Rissanen H et al. (2014) Associations of the Baltic Sea diet with cardiometabolic risk factors--a meta-analysis of three Finnish studies. Br J Nutr 112:616–626. | Irrelevant exposure |
| Kaur A, Kehinde BA, Sharma P et al. (2021) Recently isolated food-derived antihypertensive hydrolysates and peptides: A review. Food Chem 346:128719. | Irrelevant study type |
| Kellow NJ, Coughlan MT, Reid CM (2014) Metabolic benefits of dietary prebiotics in human subjects: a systematic review of randomised controlled trials. Br J Nutr 111:1147–1161. | Irrelevant exposure |
| Khan NA, Hemmelgarn B, Padwal R et al. (2007) The 2007 Canadian Hypertension Education Program recommendations for the management of hypertension: part 2 - therapy. Can J Cardiol 23:539–550. | Relevant diet-disease relationship not investigated |
| Khan NA, Hemmelgarn B, Herman RJ et al. (2008) The 2008 Canadian Hypertension Education Program recommendations for the management of hypertension: part 2 - therapy. Can J Cardiol 24:465–475. | Relevant diet-disease relationship not investigated |
| Khan NA, Hemmelgarn B, Herman RJ et al. (2009) The 2009 Canadian Hypertension Education Program recommendations for the management of hypertension: Part 2--therapy. Can J Cardiol 25:287–298. | Irrelevant study type |
| Khorraminezhad L, Rudkowska I (2021) Effect of yogurt consumption on metabolic syndrome risk factors: a narrative review. Curr Nutr Rep 10:83–92. | Irrelevant exposure |
| Kim Y, Je Y (2016) Dairy consumption and risk of metabolic syndrome: a meta-analysis. Diabet Med 33:428–440. | Relevant diet-disease relationship not investigated |
| Klonizakis M, Bugg A, Hunt B et al. (2021) Assessing the physiological effects of traditional regional diets targeting the prevention of cardiovascular disease: a systematic review of randomized controlled trials implementing Mediterranean, New Nordic, Japanese, Atlantic, Persian and Mexican dietary interventions. Nutrients 13:3034. | Irrelevant exposure |
| Koelman L, Egea Rodrigues C, Aleksandrova K (2022) Effects of dietary patterns on biomarkers of inflammation and immune responses: a systematic review and meta-analysis of randomized controlled trials. Adv Nutr 13:101–115. | Irrelevant outcome |
| Kou T, Wang Q, Cai J et al. (2017) Effect of soybean protein on blood pressure in postmenopausal women: a meta-analysis of randomized controlled trials. Food Funct 8:2663–2671. | Irrelevant exposure |
| Krittanawong C, Isath A, Hahn J et al. (2021) Mushroom Consumption and Cardiovascular Health: A Systematic Review. Am J Med 134:637-642.e2. | Irrelevant exposure |
| Kromhout D, Spaaij CJK, Goede J de et al. (2016) The 2015 Dutch food-based dietary guidelines. Eur J Clin Nutr 70:869–878. | Relevant diet-disease relationship not investigated |
| Lee M, Lee H, Kim J (2018) Dairy food consumption is associated with a lower risk of the metabolic syndrome and its components: a systematic review and meta-analysis. Br J Nutr 120:373–384. | Relevant diet-disease relationship not investigated |
| Lee YP, Puddey IB, Hodgson JM (2008) Protein, fibre and blood pressure: potential benefit of legumes. Clin Exp Pharmacol Physiol 35:473–476. | Irrelevant study type |
| Leidy HJ, Clifton PM, Astrup A et al. (2015) The role of protein in weight loss and maintenance. Am J Clin Nutr 101, Suppl:1320S–1329S. | Irrelevant study type |
| Liang W, Lee AH, Binns CW et al. (2007) Are soy foods protective against ischemic stroke? Future Neurology 2:505–511. | Irrelevant study type |
| Lovegrove JA, Hobbs DA (2016) New perspectives on dairy and cardiovascular health. Proc Nutr Soc 75:247–258. | Irrelevant study type |
| Lu M, Wan Y, Yang B et al. (2018) Effects of low-fat compared with high-fat diet on cardiometabolic indicators in people with overweight and obesity without overt metabolic disturbance: a systematic review and meta-analysis of randomised controlled trials. Br J Nutr 119:96–108. | Irrelevant exposure |
| Macedo RCO, Santos HO, Tinsley GM et al. (2020) Low-carbohydrate diets: Effects on metabolism and exercise - A comprehensive literature review. Clin Nutr ESPEN 40:17–26. | Irrelevant exposure |
| Martini LA, Wood RJ (2009) Milk intake and the risk of type 2 diabetes mellitus, hypertension and prostate cancer. Arq Bras Endocrinol Metabol 53:688–694. | Relevant diet-disease relationship not investigated |
| Massara P, Zurbau A, Glenn AJ et al. (2022) Nordic dietary patterns and cardiometabolic outcomes: a systematic review and meta-analysis of prospective cohort studies and randomised controlled trials. Diabetologia 65:2011–2031. | Irrelevant exposure |
| McCauley KM (2007) Modifying women's risk for cardiovascular disease. J Obstet Gynecol Neonatal Nurs 36:116–124. | Relevant diet-disease relationship not investigated |
| McGrane MM, Essery E, Obbagy J et al. (2011) Dairy consumption, blood pressure, and risk of hypertension: an evidence-based review of recent literature. Curr Cardiovasc Risk Rep 5:287–298. | Relevant diet-disease relationship not investigated |
| McRae MP (2016) Therapeutic benefits of l-arginine: an umbrella review of meta-analyses. J Chiropr Med 15:184–189. | Irrelevant exposure |
| Mehio Sibai A, Nasreddine L, Mokdad AH et al. (2010) Nutrition transition and cardiovascular disease risk factors in Middle East and North Africa countries: reviewing the evidence. Ann Nutr Metab 57:193–203. | Relevant diet-disease relationship not investigated |
| Messina M (2014) Soy foods, isoflavones, and the health of postmenopausal women. Am J Clin Nutr 100, Suppl 1:423S-30S. | Irrelevant study type |
| Molla MM, Ren X, Rahman E et al. (2021) Use of Chou’s 5-steps rule to study the effect of cereal dietary protein on liver and coronary heart disease prevention. Curr Nutr Food Sci 17:11–27. | Irrelevant study type |
| Naude CE, Brand A, Schoonees A et al. (2022) Low-carbohydrate versus balanced-carbohydrate diets for reducing weight and cardiovascular risk. Cochrane Database Syst Rev 1:CD013334. https://doi.org/10.1002/14651858.CD013334.pub2 | Irrelevant exposure |
| Neale EP, Batterham MJ, Tapsell LC (2016) Consumption of a healthy dietary pattern results in significant reductions in C-reactive protein levels in adults: a meta-analysis. Nutr Res 36:391–401. | Irrelevant outcome |
| Nieman KM, Anderson BD, Cifelli CJ (2021) The effects of dairy product and dairy protein intake on inflammation: a systematic review of the literature. J Am Coll Nutr 40:571–582. | Irrelevant outcome |
| Norde MM, Collese TS, Giovannucci E et al. (2021) A posteriori dietary patterns and their association with systemic low-grade inflammation in adults: a systematic review and meta-analysis. Nutr Rev 79:331–350. | Irrelevant exposure |
| Pal S, Radavelli-Bagatini S (2013) The effects of whey protein on cardiometabolic risk factors. Obes Rev 14:324–343. | Irrelevant study type |
| Papadaki A, Nolen-Doerr E, Mantzoros CS (2020) The effect of the mediterranean diet on metabolic health: a systematic review and meta-analysis of controlled trials in adults. Nutrients 12:3342. | Irrelevant exposure |
| Popolo A, Adesso S, Pinto A et al. (2014) L-Arginine and its metabolites in kidney and cardiovascular disease. Amino acids 46:2271–2286. | Relevant diet-disease relationship not investigated |
| Prentice AM (2014) Dairy products in global public health. Am J Clin Nutr 99, Suppl:1212S–1216S. | Relevant diet-disease relationship not investigated |
| Pripp AH (2008) Effect of peptides derived from food proteins on blood pressure: a meta-analysis of randomized controlled trials. Food Nutr Res 52. | Irrelevant exposure |
| Pryde MM, Kannel WB (2010) Efficacy of dietary behavior modification for preserving cardiovascular health and longevity. Cardiol Res Pract 2011:820457. | Relevant diet-disease relationship not investigated |
| Qin L-Q, Xu J-Y, Dong J-Y et al. (2013) Lactotripeptides intake and blood pressure management: a meta-analysis of randomised controlled clinical trials. Nutr Metab Cardiovasc Dis 23:395–402. | Irrelevant exposure |
| Rafique H, Dong R, Wang X et al. (2022) Dietary-nutraceutical properties of oat protein and peptides. Front Nutr 9:950400. | Irrelevant study type |
| Rahmati Najarkolaei F, Ghaffarpasand E, Gholami Fesharaki M et al. (2015) Nutrition and physical activity educational intervention on CHD risk factors: a systematic review study. Arch Iran Med 18:51–57. | Irrelevant exposure |
| Rakhmat II, Putra ICS, Wibowo A et al. (2022) Cardiometabolic risk factors in adults with normal weight obesity: a systematic review and meta-analysis. Clin Obes 12:e12523. | Irrelevant exposure |
| Ralston RA, Lee JH, Truby H et al. (2012) A systematic review and meta-analysis of elevated blood pressure and consumption of dairy foods. J Hum Hypertens 26:3–13. | Relevant diet-disease relationship not investigated |
| Rehan HS, Grover A, Hungin APS (2017) Ambiguities in the guidelines for the management of arterial hypertension. Indian perspective with a call for global harmonization. Curr Hypertens Rep 19:17. | Irrelevant exposure |
| Richard C, Cristall L, Fleming E et al. (2017) Impact of egg consumption on cardiovascular risk factors in individuals with type 2 diabetes and at risk for developing diabetes. A systematic review of randomized nutritional intervention studies. Can J Diabetes 41:453-463. | Relevant diet-disease relationship not investigated |
| Rodríguez-Monforte M, Sánchez E, Barrio F et al. (2017) Metabolic syndrome and dietary patterns: a systematic review and meta-analysis of observational studies. Eur J Nutr 56:925–947. | Irrelevant exposure |
| Roy SJ, Tanaka H (2021) Whole milk and full-fat dairy products and hypertensive risks. Curr Hypertens Rev 17:181–195. | Irrelevant study type |
| Salas-Salvadó J (2017) Dairy product consumption and risk of cardiovascular diseases. Ann Nutr Metab 71 Suppl 2:270–271. | Only abstract available |
| Santos FL, Esteves SS, da Costa Pereira A et al. (2012) Systematic review and meta-analysis of clinical trials of the effects of low carbohydrate diets on cardiovascular risk factors. Obes Rev 13:1048–1066. | Irrelevant exposure |
| Savaiano DA, Hutkins RW (2021) Yogurt, cultured fermented milk, and health: a systematic review. Nut Rev 79:599–614. | Irrelevant exposure |
| Sayon-Orea C, Martínez-González MA, Ruiz-Canela M et al. (2017) Associations between yogurt consumption and weight gain and risk of obesity and metabolic syndrome: a systematic review. Adv Nutr 8, Suppl:146S-154S. | Relevant diet-disease relationship not investigated |
| Schwingshackl L, Chaimani A, Hoffmann G et al. (2017) Impact of different dietary approaches on blood pressure in hypertensive and prehypertensive patients: protocol for a systematic review and network meta-analysis. BMJ Open 7:e014736. | Irrelevant study type |
| Schwingshackl L, Chaimani A, Schwedhelm C et al. (2019) Comparative effects of different dietary approaches on blood pressure in hypertensive and pre-hypertensive patients: a systematic review and network meta-analysis. Crit Rev Food Sci Nutr 59:2674–2687. | Relevant diet-disease relationship not investigated |
| Schwingshackl L, Hoffmann G (2014) Comparison of the long-term effects of high-fat v. low-fat diet consumption on cardiometabolic risk factors in subjects with abnormal glucose metabolism: a systematic review and meta-analysis. Br J Nutr 111:2047–2058. | Relevant diet-disease relationship not investigated |
| Schwingshackl L, Hoffmann G, Iqbal K et al. (2018) Food groups and intermediate disease markers: a systematic review and network meta-analysis of randomized trials. Am J Clin Nutr 108:576–586. | Relevant diet-disease relationship not investigated |
| Shang X, Scott D, Hodge AM et al. (2016) Dietary protein intake and risk of type 2 diabetes: results from the Melbourne Collaborative Cohort Study and a meta-analysis of prospective studies. Am J Clin Nutr 104:1352–1365. | Irrelevant outcome |
| Shanthi S, Bhagyalakshmi N, Manjula KR et al. (2021) Nutraceutical and therapeutic properties of edible super food: pumpkin seeds - a review. Int J Pharm Sci Rev Res 70:180–187. | Irrelevant study type |
| Shirani F, Salehi-Abargouei A, Azadbakht L (2013) Effects of Dietary Approaches to Stop Hypertension (DASH) diet on some risk for developing type 2 diabetes: a systematic review and meta-analysis on controlled clinical trials. Nutrition 29:939–947. | Irrelevant outcome |
| Soedamah-Muthu SS, Verberne LDM, Ding EL et al. (2012) Dairy consumption and incidence of hypertension: a dose-response meta-analysis of prospective cohort studies. Hypertension 60:1131–1137. | Relevant diet-disease relationship not investigated |
| Sohouli MH, Fatahi S, Lari A et al. (2022) The effect of paleolithic diet on glucose metabolism and lipid profile among patients with metabolic disorders: a systematic review and meta-analysis of randomized controlled trials. Crit Rev Food Sci Nutr 62:4551–4562. | Irrelevant exposure |
| Soltani S, Chitsazi MJ, Salehi-Abargouei A (2018) The effect of dietary approaches to stop hypertension (DASH) on serum inflammatory markers: A systematic review and meta-analysis of randomized trials. Clin Nutr 37:542–550. | Irrelevant outcome |
| Souza Ferreira C de, de Fátima de Sousa Fomes L, da Silva GES et al. (2015) Effect of chia seed (Salvia hispanica L.) consumption on cardiovascular risk factors in humans: a systematic review. Nutr Hosp 32:1909–1918. | Irrelevant exposure |
| Stelmach-Mardas M, Walkowiak J (2016) Dietary interventions and changes in cardio-metabolic parameters in metabolically healthy obese subjects: a systematic review with meta-analysis. Nutrients 8. | Irrelevant exposure |
| Stettler N, Murphy MM, Barraj LM et al. (2013) Systematic review of clinical studies related to pork intake and metabolic syndrome or its components. Diabetes Metab Syndr Obes 6:347–357. | Relevant diet-disease relationship not investigated |
| Tielemans SMAJ, Altorf-van der Kuil W, Engberink MF et al. (2013) Intake of total protein, plant protein and animal protein in relation to blood pressure: a meta-analysis of observational and intervention studies. J Hum Hypertens 27:564–571. | Critically low AMSTAR 2 rating |
| Tremblay A, Gilbert JA (2009) Milk products, insulin resistance syndrome and type 2 diabetes. J Am Coll Nutr 28 Suppl 1:91–102. | Irrelevant study type |
| Trouwborst I., Goossens G.H., Astrup A. et al. (2020) Men lose more weight and improve more in metabolic parameters following a low-calorie diet but are less able to maintain the improvements after 6 months of follow up compared to women. Obes Rev 21, Suppl 1 | Only abstract available |
| Turpeinen AM, Järvenpää S, Kautiainen H et al. (2013) Antihypertensive effects of bioactive tripeptides-a random effects meta-analysis. Ann Med 45:51–56. | Irrelevant exposure |
| Usinger L, Reimer C, Ibsen H (2012) Fermented milk for hypertension. Cochrane Database Syst Rev:CD008118. https://doi.org/10.1002/14651858.CD008118.pub2 | Irrelevant exposure |
| Visseren FLJ, Mach F, Smulders YM et al. (2021) 2021 ESC Guidelines on cardiovascular disease prevention in clinical practice. Eur Heart J 42:3227–3337. | Irrelevant exposure |
| Von Bibra H von, Ströhle A, St John Sutton M et al. (2017) Dietary therapy in heart failure with preserved ejection fraction and/or left ventricular diastolic dysfunction in patients with metabolic syndrome. Int J Cardiol 234:7–15. | Irrelevant outcome |
| Wang C-J, Shen Y-X, Liu Y (2016) Empirically Derived Dietary Patterns and Hypertension Likelihood: A Meta-Analysis. *Kidney* Blood Press Res 41:570–581. | Relevant diet-disease relationship not investigated |
| Wirunsawanya K, Upala S, Jaruvongvanich V et al. (2018) Whey protein supplementation improves body composition and cardiovascular risk factors in overweight and obese patients: a systematic review and meta-analysis. J Am Coll Nutr 37:60–70. | Irrelevant outcome |
| Ximenes P, Jimenez Sanchez F (2013) Leucine and glutamine as key amino acids in the prevention of cancer, rheumatism, high blood pressure and diabetes. Ann Nutr Metab 63 Suppl 1:1298. | Irrelevant exposure |
| Xiong X, Wang P, Li X et al. (2017) The effects of red yeast rice dietary supplement on blood pressure, lipid profile, and C-reactive protein in hypertension: A systematic review. Crit Rev Food Sci Nutr 57:1831–1851. | Relevant diet-disease relationship not investigated |
| Xu JY, Qin LQ, Wang PY et al. (2008) Effect of milk tripeptides on blood pressure: a meta-analysis of randomized controlled trials. Nutrition 24:933–940. | Irrelevant exposure |
| Zhang X-W, Yang Z, Li M et al. (2016) Association between dietary protein intake and risk of stroke: A meta-analysis of prospective studies. Int J Cardiol 223:548–551. | Irrelevant outcome |
| Zhang X-M, Zhang Y-B, Chi M-H (2016) Soy protein supplementation reduces clinical indices in type 2 diabetes and metabolic syndrome. Yonsei Med J 57:681–689. | Irrelevant population |
| Zhao D, Qi Y, Zheng Z et al. (2011) Dietary factors associated with hypertension. Nat Rev Cardiol 8:456–465. | Irrelevant study type |
| 2020 Dietary Guidelines Advisory Committee and Nutrition (2020) Dietary patterns and risk of cardiovascular disease: a systematic review. 2020 Dietary Guidelines Advisory Committee Projec. https://www.ncbi.nlm.nih.gov/books/NBK578519/pdf/Bookshelf_NBK578519.pdf. Accessed 30 Mar 2023 | Irrelevant exposure |
