# Supplementary material for: Dietary protein and blood pressure: an umbrella review of systematic reviews and evaluation of the evidence
Source: Eur J Nutr. 2024 Feb 20;63(4):1041–58. doi: 10.1007/s00394-024-03336-8 (PMC11139777; doi:10.1007/s00394-024-03336-8)
Supplement: Supplementary file 7 — Supplementary file7 (DOCX 23 KB) [file 394_2024_3336_MOESM7_ESM.docx]

Supplementary Material S7. Methodological quality assessment of systematic reviews using AMSTAR 2 (Critical assessment items are underlined).

| **Assessment**  **item**  **Study** | 1. Components of PICO included? | 1. Method a priori? Protocol reported? | 1. Comprehensive literature search strategy? | 1. Study selection performed in duplicate? | 1. Data extraction performed in duplicate? | 1. Number of excluded studies and corresponding reasons provided? | 1. Detailed study characteristics provided? | 1. Risk of bias assessed? | 1. Statistical heterogeneity assessed? | 1. Risk of bias considered in the discussion and interpretation? | 1. Discussion of any heterogeneity observed in the results? | 1. Publication bias investigated? | 1. Likely impact of existing publication bias discussed? | 1. Potential conflicts of interest stated? | Number of critical weaknesses | Number of non-critical weaknesses | **Methodological quality** |
| --- | --- | --- | --- | --- | --- | --- | --- | --- | --- | --- | --- | --- | --- | --- | --- | --- | --- |
| Bryant 2022 | Yes | Yes | Yes | No | No | Yes | Yes | Yes | No MA | Yes | No MA | No MA | No MA | No | 0 | 3 | **moderate** |
| Hengeveld 2022 | Yes | No | Yes | No | No | Yes | Yes | Yes | No MA | No | No MA | No MA | No MA | No | 0 | 5 | **moderate** |
| Mohammadifard 2021 | Yes | Yes | Yes | Yes | No | Yes | Yes | No | Yes | No | Yes | Yes | Yes | Yes | 1 | 2 | **low** |
| Mosallanezhad 2021 | Yes | Yes | Yes | Yes | No | Yes | No | Yes | No MA | Yes | No MA | No MA | No MA | Yes | 0 | 2 | **moderate** |
| Vogtschmidt 2021 | Yes | Yes | Yes | Yes | Yes | Yes | Yes | Yes | Yes | No | Yes | Yes | Yes | Yes | 0 | 1 | **high** |
| Lonnie 2020 | Yes | Yes | Yes | Yes | No | Yes | Yes | No | No MA | No | No MA | No MA | No MA | No | 1 | 3 | **low** |
| Mousavi 2020 | Yes | Yes | Yes | Yes | Yes | Yes | No | Yes | Yes | Yes | No | Yes | Yes | Yes | 0 | 2 | **moderate** |
| Badely 2019 | Yes | Yes | Yes | Yes | Yes | Yes | Yes | No | Yes | No | No | Yes | Yes | Yes | 1 | 2 | **low** |
| Chalvon-Demersay 2017 | Yes | Yes | Yes | No | No | Yes | Yes | Yes | No MA | Yes | No | No MA | No MA | Yes | 0 | 3 | **moderate** |
| Hidayat 2017 | Yes | Yes | Yes | Yes | Yes | Yes | Yes | Yes | Yes | Yes | Yes | Yes | Yes | Yes | 0 | 0 | **high** |
| Clifton 2014 | Yes | Yes | Yes | No | Yes | Yes | Yes | Yes | Yes | No | No | Yes | Yes | No | 0 | 4 | **moderate** |
| Pedersen 2013 | Yes | Yes | Yes | Yes | No | Yes | Yes | Yes | No MA | No | No MA | No MA | No MA | Yes | 0 | 2 | **moderate** |
| Schwingshackl 2013 | Yes | Yes | Yes | Yes | Yes | Yes | Yes | Yes | Yes | Yes | No | Yes | Yes | Yes | 0 | 1 | **high** |
| Rebholz 2012 | Yes | Yes | Yes | Yes | Yes | Yes | Yes | No | Yes | Yes | Yes | Yes | Yes | Yes | 1 | 0 | **low** |
| Santesso 2012 | Yes | Yes | Yes | Yes | Yes | Yes | Yes | Yes | Yes | Yes | Yes | Yes | Yes | Yes | 0 | 0 | **high** |
| Wycherley 2012 | Yes | No | Yes | Yes | No | Yes | No | Yes | Yes | No | Yes | No | No | Yes | 1 | 5 | **low** |
| Tielemans 2013 | Yes | No | No | No | Yes | Yes | Yes | No | Yes | No | Yes | No | No | Yes | 3 | 4 | **critically low** |
| Dong 2011 | Yes | No | No | No | Yes | No | Yes | Yes | Yes | No | Yes | Yes | Yes | Yes | 2 | 3 | **critically low** |
| Altorf-van der Kuil 2010 | Yes | No | No | Yes | No | Yes | Yes | No | No MA | Yes | No | No MA | No MA | Yes | 2 | 3 | **critically low** |

The assessment items are provided as shortened versions. The full questionnaire is provided in Supplementary Material S3.

High rating = no critical weakness with no or one non-critical weakness: the systematic review provides an accurate and comprehensive summary of the results of the available studies that address the question of interest. Moderate rating = no critical weakness with more than one non-critical weakness: the systematic review has more than one weakness but no critical flaws. It may provide an accurate summary of the results of the available studies that were included in the review. Low rating = one critical weakness with or without non-critical weaknesses: the review has a critical flaw and may not provide an accurate and comprehensive summary of the available studies that address the question of interest. Critically low rating = more than one critical weakness with or without non-critical weaknesses: the review has more than one critical flaw and should not be relied on to provide an accurate and comprehensive summary of the available studies. Shea BJ, Reeves BC, Wells G, Thuku M, Hamel C, Moran J, et al. AMSTAR 2: a critical appraisal tool for systematic reviews that include randomised or non-randomised studies of healthcare interventions, or both. BMJ. 2017 Sep;358(j4008):1-8.

AMSTAR 2: A Measurement Tool to Assess Systematic Reviews 2; MA: meta-analysis;
