# Supplementary material for: Dietary protein and blood pressure: an umbrella review of systematic reviews and evaluation of the evidence
Source: Eur J Nutr. 2024 Feb 20;63(4):1041–58. doi: 10.1007/s00394-024-03336-8 (PMC11139777; doi:10.1007/s00394-024-03336-8)
Supplement: Supplementary file 8 — Supplementary file8 (DOCX 37 KB) [file 394_2024_3336_MOESM8_ESM.docx]

Supplementary Material S8. Outcome-specific certainty of evidence assessment of included systematic reviews using NutriGrade.

A) SR with MA of RCTs

| Systematic review | Exposure | Outcome | 1. Risk of bias | 2. Precision | 3. Heterogeneity | 4. Directness | 5. Publication bias | 6. Funding bias | 7. Study design | Overall score | **Outcome-specific certainty of evidence** |
| --- | --- | --- | --- | --- | --- | --- | --- | --- | --- | --- | --- |
| Rebholz 2012 | Total protein | SBP | 1 | 1 | 0.8 | 0 | 1 | 1 | 2 | 6.8 | **moderate** |
| Rebholz 2012 |  | DBP | 1 | 1 | 0.8 | 0 | 1 | 1 | 2 | 6.8 | **moderate** |
| Santesso 2012 |  | SBP | 1 | 1 | 0.8 | 0 | 1 | 0 | 2 | 5.8 | **low** |
| Santesso 2012 |  | DBP | 1 | 1 | 0.8 | 0 | 1 | 0 | 2 | 5.8 | **low** |
| Wycherley 2012 |  | SBP | 1 | 0 | 0 | 0 | 0 | 1 | 2 | 4 | **low** |
| Wycherley 2012 |  | DBP | 1 | 0 | 0 | 0 | 0 | 1 | 2 | 4 | **low** |
| Schwingshackl 2013 |  | SBP | 1.5 | 0 | 0.4 | 0 | 0.5 | 1 | 2 | 5.4 | **low** |
| Schwingshackl 2013 |  | DBP | 1.5 | 0 | 0.8 | 0 | 0.5 | 1 | 2 | 5.8 | **low** |
| Clifton 2014 |  | SBP | 0 | 1 | 0.8 | 0 | 1 | 0 | 2 | 4.8 | **low** |
| Clifton 2014 |  | DBP | 0 | 1 | 0.6 | 0 | 1 | 0 | 2 | 4.6 | **low** |
| Vogtschmidt 2021 |  | SBP (SMD) | 1 | 1 | 0.8 | 0 | 1 | 0 | 2 | 5.8 | **low** |
| Vogtschmidt 2021 |  | DBP (SMD) | 1 | 0 | 0.8 | 0 | 1 | 0 | 2 | 4.8 | **low** |
| Vogtschmidt 2021 |  | SBP (WMD) | 1 | 1 | 0.8 | 0 | 1 | 0 | 2 | 5.8 | **low** |
| Rebholz 2012 | Animal protein | SBP | 1 | 1 | 0.8 | 0 | 1 | 1 | 2 | 6.8 | **moderate** |
| Rebholz 2012 |  | DBP | 1 | 1 | 0.8 | 0 | 1 | 1 | 2 | 6.8 | **moderate** |
| Hidayat 2017 |  | SBP | 1 | 1 | 0.4 | 0 | 0.5 | 0.5 | 2 | 5.4 | **low** |
| Hidayat 2017 |  | DBP | 1 | 1 | 0.4 | 0 | 0.5 | 0.5 | 2 | 5.4 | **low** |
| Badely 2019 |  | SBP | 0 | 1 | 0.6 | 0 | 0.5 | 1 | 2 | 5.1 | **low** |
| Badely 2019 |  | DBP | 0 | 1 | 0.6 | 0 | 0.5 | 1 | 2 | 5.1 | **low** |
| Rebholz 2012 | Plant protein | SBP | 1 | 1 | 0.8 | 0 | 1 | 1 | 2 | 6.8 | **moderate** |
| Rebholz 2012 |  | DBP | 1 | 1 | 0.8 | 0 | 1 | 1 | 2 | 6.8 | **moderate** |
| Mohammadifard 2021 |  | SBP | 0 | 0 | 0 | 0 | 0 | 1 | 2 | 3 | **very low** |
| Mohammadifard 2021 |  | DBP | 0 | 0 | 0 | 0 | 0 | 1 | 2 | 3 | **very low** |
| Rebholz 2012 | Animal vs. plant protein | SBP | 1 | 0 | 0.8 | 0 | 0.5 | 1 | 2 | 5.3 | **low** |
| Rebholz 2012 |  | DBP | 1 | 0 | 0.8 | 0 | 0.5 | 1 | 2 | 5.3 | **low** |

Abbreviations: DBP: diastolic blood pressure; MA: meta-analysis/meta-analyses; RCT: randomised controlled trial; SBP: systolic blood pressure; SMD: standardised mean difference; SR: systematic review; WMD: weighted mean difference

Categorisation of the outcome-specific certainty of evidence: 0-3.99: very low; 4-5.99: low; 6-7.99: moderate; ≥8: high

B) SR with MA of cohort studies

| Systematic review | Exposure | Outcome | 1. Risk of bias | 2. Precision | 3. Heterogeneity | 4. Directness | 5. Publication bias | 6. Funding bias | 7. Effect size | 8. Dose-response | Overall score | **Outcome-specific certainty of evidence** |
| --- | --- | --- | --- | --- | --- | --- | --- | --- | --- | --- | --- | --- |
| Mousavi 2020 | Total protein | hypertension | 2 | 1 | 1 | 1 | 0 | 1 | 0 | 0 | 6 | **moderate** |
| Mousavi 2020 | Animal protein | hypertension | 2 | 1 | 1 | 1 | 0 | 1 | 0 | 0 | 6 | **moderate** |
| Mousavi 2020 | Plant protein | hypertension | 2 | 0 | 1 | 1 | 0 | 1 | 0 | 1 | 6 | **moderate** |

Abbreviations: MA: meta-analysis/meta-analyses; SR: systematic review

Categorisation of the outcome-specific certainty of evidence: 0-3.99: very low; 4-5.99: low; 6-7.99: moderate; ≥8: high

C) SR without MA of RCTs

| Systematic review | Exposure | Outcome | 1. Risk of bias | 2. Precision | 3. Heterogeneity | 4. Directness | 5. Funding bias | 6. Study design | Overall score | **Outcome-specific certainty of evidence** |
| --- | --- | --- | --- | --- | --- | --- | --- | --- | --- | --- |
| Pedersen 2013 | Total protein | SBP | 1 | 0 | 1 | 0 | 1 | 2 | 5 | **low** |
| Pedersen 2013 |  | DBP | 1 | 0 | 1 | 0 | 1 | 2 | 5 | **low** |
| Lonnie 2020 |  | SBP | 1 | 0 | 0 | 0 | 0 | 2 | 3 | **very low** |
| Lonnie 2020 |  | DBP | 1 | 0 | 0 | 0 | 0 | 2 | 3 | **very low** |
| Hengeveld 2022 |  | SBP | 1 | 0 | 1 | 0 | 0.5 | 2 | 4.5 | **low** |
| Hengeveld 2022 |  | DBP | 1 | 0 | 1 | 0 | 0.5 | 2 | 4.5 | **low** |
| Mosallanezhad 2021 | Plant protein | SBP | 2.5 | 1 | 0 | 0 | 1 | 2 | 6.5 | **moderate** |
| Mosallanezhad 2021 |  | DBP | 2.5 | 1 | 0 | 0 | 1 | 2 | 6.5 | **moderate** |
| Chalvon-Demersay  2017 | Animal vs. plant protein | SBP | 2 | 1 | 1 | 0 | 0 | 2 | 6 | **moderate** |
| Chalvon-Demersay  2017 |  | DBP | 2 | 1 | 1 | 0 | 0 | 2 | 6 | **moderate** |
| Lonnie 2020 |  | SBP | 1 | 0 | 1 | 0 | 0 | 2 | 4 | **low** |
| Lonnie 2020 |  | DBP | 1 | 0 | 1 | 0 | 0 | 2 | 4 | **low** |
| Bryant 2022 |  | SBP | 2 | 0 | 1 | 0 | 0 | 2 | 5 | **low** |
| Bryant 2022 |  | DBP | 2 | 0 | 1 | 0 | 0 | 2 | 5 | **low** |

Abbreviations: DBP: diastolic blood pressure; MA: meta-analysis/meta-analyses; RCT: randomised controlled trial; SBP: systolic blood pressure; SR: systematic review

Categorisation of the outcome-specific certainty of evidence: 0-3.49: very low; 3.5-5.49: low; 5.5-6.99: moderate; ≥7: high

D) SR without MA of cohort studies

| Systematic review | Exposure | Outcome | 1. Risk of bias | 2. Precision | 3. Heterogeneity | 4. Directness | 5. Funding bias | 6. Effect size | Overall score | **Outcome-specific certainty of evidence** |
| --- | --- | --- | --- | --- | --- | --- | --- | --- | --- | --- |
| Pedersen 2013 | Total protein | hypertension | 1 | 1 | 1 | 1 | 1 | 0 | 5 | **moderate** |
| Pedersen 2013 |  | SBP | 1 | 0 | 1 | 0 | 1 | 0 | 3 | **low** |
| Pedersen 2013 |  | DBP | 1 | 0 | 1 | 0 | 1 | 0 | 3 | **low** |
| Pedersen 2013 | Animal protein | hypertension | 1 | 1 | 1 | 1 | 1 | 0 | 5 | **moderate** |
| Pedersen 2013 |  | SBP | 1 | 0 | 1 | 0 | 1 | 0 | 3 | **low** |
| Pedersen 2013 |  | DBP | 1 | 0 | 1 | 0 | 1 | 0 | 3 | **low** |
| Chalvon-Demersay 2017 |  | SBP | 2 | 1 | 1 | 0 | 0 | 0 | 4 | **low** |
| Chalvon-Demersay 2017 |  | DBP | 2 | 1 | 1 | 0 | 0 | 0 | 4 | **low** |
| Pedersen 2013 | Plant protein | hypertension | 1 | 1 | 1 | 1 | 1 | 1 | 6 | **high** |
| Pedersen 2013 |  | SBP | 1 | 0 | 1 | 0 | 1 | 1 | 4 | **low** |
| Pedersen 2013 |  | DBP | 1 | 0 | 1 | 0 | 1 | 1 | 4 | **low** |
| Chalvon-Demersay 2017 |  | SBP | 2 | 1 | 1 | 0 | 0 | 1 | 5 | **moderate** |
| Chalvon-Demersay 2017 |  | DBP | 2 | 1 | 1 | 0 | 0 | 1 | 5 | **moderate** |

Abbreviations: DBP: diastolic blood pressure; MA: meta-analysis/meta-analyses; SBP: systolic blood pressure; SR: systematic review

Categorisation of the outcome-specific certainty of evidence: 0-2.99: very low; 3-4.49: low; 4.5-5.99: moderate; ≥6: high
